# Breakthroughs in modern cancer therapy and elusive cardiotoxicity: Critical research‐practice gaps, challenges, and insights

**DOI:** 10.1002/med.21463

**Published:** 2017-09-01

**Authors:** Ping‐Pin Zheng, Jin Li, Johan M Kros

**Affiliations:** ^1^ Cardio‐Oncology Research Group Erasmus Medical Center Rotterdam the Netherlands; ^2^ Department of Pathology Erasmus Medical Center Rotterdam the Netherlands; ^3^ Department of Oncology Shanghai East Hospital, Tongji University School of Medicine Shanghai China

**Keywords:** cancer therapy‐induced cardiotoxicity, chimeric antigen receptor (CAR) T‐cell therapy, immune checkpoint inhibitors, molecularly targeted therapeutics, research‐practice gaps

## Abstract

To date, five cancer treatment modalities have been defined. The three traditional modalities of cancer treatment are surgery, radiotherapy, and conventional chemotherapy, and the two modern modalities include molecularly targeted therapy (the fourth modality) and immunotherapy (the fifth modality). The cardiotoxicity associated with conventional chemotherapy and radiotherapy is well known. Similar adverse cardiac events are resurging with the fourth modality. Aside from the conventional and newer targeted agents, even the most newly developed, immune‐based therapeutic modalities of anticancer treatment (the fifth modality), e.g., immune checkpoint inhibitors and chimeric antigen receptor (CAR) T‐cell therapy, have unfortunately led to potentially lethal cardiotoxicity in patients. Cardiac complications represent unresolved and potentially life‐threatening conditions in cancer survivors, while effective clinical management remains quite challenging. As a consequence, morbidity and mortality related to cardiac complications now threaten to offset some favorable benefits of modern cancer treatments in cancer‐related survival, regardless of the oncologic prognosis. This review focuses on identifying critical research‐practice gaps, addressing real‐world challenges and pinpointing real‐time insights in general terms under the context of clinical cardiotoxicity induced by the fourth and fifth modalities of cancer treatment. The information ranges from basic science to clinical management in the field of cardio‐oncology and crosses the interface between oncology and onco‐pharmacology. The complexity of the ongoing clinical problem is addressed at different levels. A better understanding of these research‐practice gaps may advance research initiatives on the development of mechanism‐based diagnoses and treatments for the effective clinical management of cardiotoxicity.

## 1 INTRODUCTION

Currently available cancer treatments include the traditional surgery, radiotherapy, and conventional chemotherapy approaches and have been extended with two new modalities in recent decades: molecularly targeted therapy (MTT) and immunotherapy. Cardiac toxicities associated with conventional chemotherapy and radiotherapy are well known, and to a certain degree, the cellular and molecular mechanisms leading to cardiotoxicity have been discovered. Similar adverse cardiac events are encountered when applying MTTs, which represent a new generation of anticancer drugs.^1^, ^2^, ^3^, ^4^, ^5^, ^6^, ^7^, ^8^, ^9^, ^10^, ^11^ The MTTs that have received US FDA approval since their initiation in 2001 are listed in Tables 1, 2, and 3. In addition, the recently developed immune‐based therapeutic modalities, e.g., immune checkpoint inhibitors (ICIs)^12^, ^13^, ^14^, ^15^, ^16^, ^17^, ^18^, ^19^, ^20^, ^21^, ^22^, ^23^, ^24^, ^25^, ^26^, ^27^, ^28^, ^29^ and chimeric antigen receptor (CAR) T‐cell therapy (CART),^29^, ^30^, ^31^, ^32^, ^33^, ^34^, ^35^, ^36^, ^37^, ^38^, ^39^, ^40^, ^41^, ^42^, ^43^, ^44^ have also raised cardiovascular concerns in patients, including lethal cardiotoxicity (Table 3). Cardiotoxicity reflects functional, structural or a combination of both types of damage to the heart by various detrimental environmental insults,^45^ e.g., conventional chemotherapy, radiotherapy, MTT, immunotherapy, and toxins. Cardiotoxicity manifests as electrophysiological disorder of the heart (cardiac dysfunction) and a spectrum of myocardial damage (cardiomyopathy), with heart failure as the most severe consequence. The clinical features are demonstrated by a wide range of cardiovascular manifestations or events, including bradycardia, tachycardia, cardiomyopathy, widened pulse pressure, hypotension, arrhythmias, decreased left ventricular ejection fraction (LVEF), troponinemia, QT prolongation, myocarditis, myocardial infarction, pericarditis, acute coronary syndromes, and congestive heart failure.^20^, ^46^, ^47^, ^48^, ^49^ The clinical scenarios derived from these novel anticancer approaches reflect new challenges to medical, pharmacological, and research communities because the cardiotoxicity from new clinical entities are clinically and mechanistically different from the cardiotoxicity resulting from traditional chemotherapeutic agents. Cardiotoxicity caused by these novel agents or modalities are diverse, and many specific mechanisms of individual compounds or modalities underlying this toxicity remain to be elucidated.^50^ Some commonly conceptualized mechanisms underlie toxicity, including on‐ and off‐target toxicity, production of toxic metabolites, harmful immune responses, unpredictable specificities of targeted tumor antigens, tumor lysis symptoms, cytokine release syndrome, T‐cell receptor (TCR) mispairing, TCR cross‐reactivity, and idiosyncratic mechanisms.^51^, ^52^, ^53^, ^54^, ^55^, ^56^

**Table 1:** Approved molecularly targeted therapeutics (non‐mAbs) in oncology and reported clinical cardiotoxicity/cardiovascular events from 2001 to May 2017

Generic/Trade Name	Approval Year	Major Known Targets	Disease Indications	Cardiotoxicity/cardiovascular events
Gleevec (Imatinib mesylate)	2001,2002	ABL1‐2, PDGFR, KIT	Ph+CML, GIST	SR1‐13
Velcade (Bortezomib)	2003	Proteasome	Multiple myeloma	SR4,10,12,14‐19
Iressa (Gefitinib)	2003	EGFR	NSCLC	SR20,21
Tarceva (Erlotinib)	2004	EGFR	NSCLC	SR22‐26
Nexavar (sorafenib)	2005	KIT, PDGFR‐B, RET, BRAF, VEGFR2/3, FLT‐3	RCC, HCC, GIST	SR3,7,9,10,16,27‐29
Sprycel (Dasatinib)	2006	ABL1, SRC	CML	SR3,4,7,8,10,12,16, 30‐32
Sutent (Sunitinib malate)	2006, 2011	VEGFR, PDGFR, KIT, FLT3, RET, CSF‐1R	RCC, GIST, PNT	SR1‐4,7‐10,12,16,27, 30,33‐36
Zolinza (Vorinostat)	2006	HDAC	Cutaneous T‐cell lymphoma	SR7,10,30,37
Tasigna (Nilotinib)	2007	ABL1, KIT, PDGF	Ph(+) CML	SR3,7,10,12,16,38
Torisel (Temsirolimus)	2007	mTOR	ARCC	SR10,16,39
Tykerb (Lapatinib)	2007	EGFR, ERBB2	Breast cancer	SR3,4,7,8,10,16,25, 40‐44
Istodax (Romidepsin)	2009	HDAC	Cutaneous T‐cell lymphoma	SR7,10,45‐50
Afinitor (Everolimus)	2009, 2011, 2012	mTOR	RCC, APNT, HER2(‐) breast cancer	SR10,16,25,51
Votrient (Pazopanib)	2009, 2012	VEGFR1/2/3	RCC, soft tissue sarcoma	SR7,10,12,16,52
Xalkori (Crizotinib)	2011	MET, ALK	ALK(+) NSCLC	SR7,12,16,53‐55
Vandetanib (Vandetanib)	2011	VEGFR, EGFR	Thyroid cancer	SR7,12,16,56,57
Zelboraf (Vemurafenib)	2011	BRAF	BRAF(+) melanoma	SR12,28,58‐63
JAKAFI (Ruxolitinib)	2011	JAK2	Myelofibrosis	SR64,65
Kyprolis (Carfilzomib)	2012	Proteasome	Multiple myeloma	SR12,16,66‐70
Bosulif (Bosutinib)	2012	SRC, ABL1	Ph(+) chronic myelogenous leukemia	SR7,71
Stivarga (Regorafenib)	2012, 2013	VEGFR2, TIE2	Metastatic colorectal cancer, GIST	SR16,28,72
Cabometyx (Cabozantinib)	2012, 2016	RET, MET, VEGFR1/2/3, KIT, TRKB, FLT‐3, AXL, TIE‐2, TYRO3, MER	Metastatic medullary thyroid cancer, ARCC	SR7,16,73,74
Zaltrap (ziv‐aflibercept)	2012	PIGF, VEGF‐A	Metastatic colorectal cancer	SR7,10,16,75
Erivedge (Vismodegib)	2012	Hedgehog (Hh) signaling pathway	Basal cell carcinoma	Reports not found yet
Inlyta (Axitinib)	2012	VEGFR1/2/3	ARCC	SR16,76,77
Iclusig (Ponatinib)	2012	Abl, Src	CML, Ph(+) ALL	SR7,12,16,78
Gilotrif (Afatinib)	2013	EGFR, ERBB2, ERBB4	Metastatic NSCLC with EGFR mutations	SR43,79
Tafinlar (Dabrafenib)	2013	BRAF	Melanoma	SR7,80
Mekinist (Trametinib)	2013	MEK1, MEK2	Melanoma with BRAF V600E or V600K mutations	SR7,12,16,81‐83
Imbruvica (ibrutinib)	2013, 2014	Bruton's kinase (BTK)	Mantle cell lymphoma, CLL	SR16,84
Zykadia (Ceritinib)	2014	ALK	ALK(+) metastatic NSCLC	SR12,16,85
Zydelig (Idelalisib)	2014	PI3K delta	CLL, follicular B‐cell NHL, SLL	SR86
Lynparza (Olaparib)	2014	PARP	BRCA mutated advanced ovarian cancer	SR87
Beleodaq (Belinostat)	2014	HDAC	Relapsed or refractory peripheral T‐cell lymphoma	SR88,89
Alecensa (Alectinib)	2015	ALK, RET	ALK‐positive, metastatic NSCLC	SR90
Cotellic (Cobimetinib)	2015	MAPK	BRAF V600E or V600K melanoma	SR91
Lenvima (Lenvatinib)	2015, 2016	VEGFR1/2/3, FGFR1/2/3, PDGFRα, KIT, RET	Thyroid cancer, ARCC	SR16,92
Tagrisso (Osimertinib)	2015	EGFR	EGFR T790M mutation positive NSCLC	SR93
Ibrance (Palbociclib)	2015	CDK4/6	ER(+), HER2(‐) breast cancer	SR94,95
Odomzo (Sonidegib)	2015	Hedgehog pathway (Smoothened)	Locally advanced basal cell carcinoma	Reports not found yet
Farydak (Panobinostat)	2015	HDAC	Multiple myeloma	SR47,50,96,97
Ninlaro (Ixazomib)	2015	Beta 5 subunit of the 20S proteasome	Multiple myeloma	SR98
Lonsurf (Trifluridine and tipiracil)	2015	Nucleoside metabolic and thymidine phosphorylase	Metastatic colorectal cancer	SR99
Onivyde(Irinotecan liposome injection)	2015	Topoisomerase	Metastatic pancreatic cancer	SR100
Venclexta (Venetoclax)	2016	BCL‐2	CLL with 17p deletion	SR101
Rubraca (Rucaparib)	2016	PARP	Advanced ovarian cancer with BRCA mutation	Reports not found
Alunbrig (brigatinib)	2017	ALK	Advanced ALK‐positive metastatic NSCLC	SR163, SR164
Rydapt (midostaurin)	2017	FLT3	FLT3 positive acute myeloid leukemia and mastocytosis	SR165
Zejula (niraparib)	2017	Poly(ADP‐ribose) polymerase (PARP) inhibitor	Recurrent epithelial ovarian, fallopian tube, etc	SR166
Kisqali (ribociclib)	2017	Cyclin‐dependent kinase (CDK) 4 and 6	Breast cancer	SR168

**Abbreviations**: NSCLC = Non‐small‐cell lung carcinoma; RCC = Renal cell carcinoma; CML = chronic myelogenous leukemia; GISTs = Gastrointestinal stromal tumors; ARCC = Advanced renal cell carcinoma; RCC = Renal cell carcinoma; PNT = Pancreatic neuroendocrine tumors; APNT = Advanced pancreatic neuroendocrine tumors; Ph(+) = Philadelphia (+); CLL = Chronic lymphoblastic leukemia; HDAC = Histone deacetylase; PARP = Poly(ADP‐ribose) polymerase; ALK = Anaplastic lymphoma kinase; CSF‐1R = Colony stimulating factor receptor Type 1; FLT3 = Fms‐like tyrosine kinase‐3; PDGFR = Platelet‐derived growth factor receptors; PI3K = Phosphoinositide‐3 kinase; HCC = Hepatocellular cancer; MAPK = Mitogen‐activated protein kinase; EGFR = Epidermal growth factor receptor; VEGFR = Vascular endothelial growth factor receptor; FGFR = Fibroblast growth factor receptors; PIFG = Placental Growth Factor;JAK2 = Janus kinase 2; TRKB = Tropomyosin receptor kinase B; TYRO3 = Tyrosine‐protein kinase receptor; mTOR = Mammalian target of rapamycin; SLL = Small lymphocytic lymphoma; SR = Supplemental references.

**Table 2:** Approved therapeutic monoclonal antibodies (non‐ICIs) in oncology and the reported clinical cardiotoxicity/cardiovascular events from 2001 to May 2017

Generic/Trade Names	FDA Approval	Antigens	Disease Indications	Cardiotoxicity/Cardiovascular Events
Campath (Alemtuzumab)	2001	CD52	B‐CLL	SR102‐105
Zevalin (Ibritumomab tiuxetan)	2002	CD20	NHL	SR14,102,106
Bexxar (Tositumomab)	2003	CD20	NHL, follicular NHL	SR107,108
Erbitux (Cetuximab)	2004	EGFR	EGFR(+) MBC	SR10,102,109
Avastin (Bevacizumab)	2004, 2009	VEGFR	Colorectal Cancer, RCC	SR4,7,9,10,16,25,27,40,102,103,110
Vectibix (Panitumumab)	2006	EGFR	Colorectal cancer	SR111
Rituxan (Rituximab)	2006, 2010, 2011, 2012	CD20	Follicular B‐cell, CD20 (+) NHL, CLL	SR11,102,103,112,113
Arzerra (Ofatumumab)	2009	CD20	CLM	SR114
Herceptin (Trastuzumab)	2010, 2013	HER2	Gastric cancer, HER2(+) MBC	SR4,7‐10,12,16,25,27,31,35,40,42,102,103,115‐119
Xgeva (Denosumab)	2010, 2013	RANKL	Bone metastases from solid tumors, GCT	SR120
Perjeta (Pertuzumab)	2012	ERBB2	HER2+ MBC	SR7,16,25,43,102,121‐123
Adcetris (Brentuximab vedotin)	2011	CD30	HL, ALCL	SR102,103,124
Kadcyla (Trastuzumab emtansine)	2013	HER2	HER2 (+) MBC	SR16,43,102,103,125
Gazyva (Obinutuzumab)	2013	CD20	Chronic lymphocytic leukemia,	SR102,126,127
Cyramza (Ramucirumab)	2014	VEGFR	Gastric cancer	SR16,84,102,128
Darzalex (Daratumumab)	2015	CD38	Multiple myeloma	SR129
Empliciti (Elotuzumab)	2015	SLAMF7	Multiple myeloma	SR130
Portrazza (Necitumumab)	2015	EGFR	Metastatic SNSCLC	SR131
Unituxin (Dinutuximab)	2015	Glycolipid GD2	Neuroblastoma	SR132
Lartruvo (Olaratumab)	2016	PDGFRα	Soft tissue sarcoma	SR133

**Abbreviations**: NHL = Non‐Hodgkin lymphoma; HL = Hodgkin lymphoma; GCT = Giant cell tumor of bone; SLAMF7 = Signaling Lymphocytic Activation Molecule Family member 7; CLM = Chronic lymphocytic leukemia; RANKL = Receptor activator of nuclear factor kappa‐B ligand; PDGFRα = Platelet‐derived growth factor receptor alpha; IL‐6 = Interleukin 6; Non‐ICIs = Non‐immune checkpoint inhibitors; RCC = Renal cell carcinoma; B‐CLL = B‐cell chronic lymphocytic leukemia; PDGFR = Platelet‐derived growth receptor; CLL = Chronic lymphocytic leukemia; SNSCLC = Squamous non‐small cell lung cancer; MBC = Metastatic breast cancer; ALCL = Anaplastic large cell lymphoma; SR = Supplemental references.

**Table 3:** Immune checkpoint inhibitors and CART in oncology and the reported clinical cardiotoxicity/cardiovascular events from 2011 to May 2017

Generic/Trade Name	Approval Year	Category	Antigens	Disease Indications	Cardiotoxicity/Cardiovascular Events
Yervoy (Ipilimumab)	2011	ICI	CTLA‐4	Metastatic melanoma	SR16,134‐136
Opdivo (Nivolumab)	2014, 2015, 2016	ICI	PD‐1	Metastatic melanoma, metastatic SNSCLC, ARCC	SR16,135‐140
Keytruda (Pembrolizumab)	2014, 2015, 2016	ICI	PD‐1	Head and neck SCC	SR16,135‐137,141‐143
Tecentriq (Atezolizumab)	2016	ICI	PD‐L1	Urothelial carcinoma and metastatic NSCLC	SR144
Bavencio (avelumab)	2017	ICI	PD‐L1	Merkel cell carcinoma	SR160, SR161, SR167
Imfinzi (durvalumab)	2017	ICI	PD‐L1	Advanced or metastatic urothelial carcinoma	SR162, SR167
CART	Not yet	CART	CD19, MAGE‐A3	Hematopoietic malignancies, melanoma	SR145‐159

**Abbreviations**: CTLA‐4 = Cytotoxic T‐lymphocyte‐associated antigen 4; ICIs = Immune checkpoint inhibitors; ARCC = Advanced renal cell carcinoma; CHL = Classical Hodgkin lymphoma; SNSCLC = Metastatic squamous non‐small cell lung cancer; SCC = Squamous cell carcinoma; NSCLC = Non‐small cell lung cancer; CART = Chimeric antigen receptor T cell therapy; SR = Supplemental references.

A primary goal of targeted therapy or more advanced immune‐based modalities is to kill cancer cells more specifically than traditional treatment modalities while maintaining an acceptable level of side effects and quality of life. Unfortunately, the newer targeted agents or modalities exhibit a similar frequency and severity of toxicities as traditional cytotoxic agents do, albeit with differences in preference for the organs/tissues that are involved.^57^ Many unanticipated short‐ and long‐term adverse effects on multiple organs/tissues have shown the limitations of the new therapies,^7^, ^30^, ^56^, ^57^, ^58^, ^59^ although MTTs have improved the overall survival of cancer patients over the past two decades,^57^, ^60^, ^61^ and advanced immunotherapies are promising to improve the outcomes of certain types of cancers (ICIs^62^, ^63^, ^64^, ^65^, ^66^, ^67^, ^68^, ^69^, ^70^ and CART^71^, ^72^, ^73^, ^74^). Cardiac complications associated with cancer treatment represent unresolved and potentially life‐threatening conditions in cancer survivors. As a consequence, morbidity and mortality related to cardiac complications now threaten to offset some of the favorable benefits of these novel cancer treatments in cancer‐related survival and strongly impact the quality of life regardless of the oncologic prognosis.^75^, ^76^, ^77^ Additionally, effective clinical management remains quite challenging because no evidence‐based approaches are currently available for the effective monitoring and treatment of these patients.^78^, ^79^ This information is lacking because the risk of cardiovascular toxicity greatly differs from one treatment to another according to the targeted pathways or antigens and the presence of co‐morbid conditions.^75^, ^80^ Because the novel drugs or modalities are highly cost‐intensive,^81^, ^82^, ^83^, ^84^ cardiotoxicity may add additional costs without improving the outcome of a life‐limiting illness with a generally unpredictable occurrence and uncertain cure.^8^, ^54^, ^85^, ^86^ Clinical cardiotoxicity challenges not only sustainable cancer survivorship but also precision oncology/medicine and affordable healthcare. Cost savings are a major incentive for the adoption of biosimilars, and some biosimilars have been developed and used in clinical oncology trials.^87^, ^88^ Biosimilars refer to biologic products that demonstrate no clinically meaningful differences in terms of quality attributes, efficacy, safety, and immunogenicity compared with an existing licensed, originator biologic.^89^ The incorporation of biosimilars into healthcare systems worldwide may result in a 30–45% cost savings.^87^ A strategy to prevent clinical cardiotoxicity will have significant impacts on the overall prognosis and survival of cancer patients. This strategy is part of the field of cardiovascular safety, with multifaceted aspects at multiple levels involving improvement of preclinical models for the study of chemotherapy‐induced cardiotoxicity,^90^ appropriate preapproval investigations and monitoring during clinical practice.^91^ In addition, it is important to realize that the approval decision following preapproval clinical trials does not necessarily represent a singular moment of clarity about the risks and benefits associated with a drug; thus, continuous post‐marketing surveillance and vigilance for cardiotoxicity is required.^91^ The research gaps in novel cancer treatment‐related cardiotoxicity range from basic, translational, clinical, and epidemiologic sciences to cancer patients, medical professionals, research communities, pharmaceutical industries, regulatory bodies, and research funding agencies. In response to ongoing clinical challenges, “cardio‐oncology” or “onco‐cardiology” represents a new multidisciplinary discipline in recent decades^92^, ^93^ and is a newer frontier in clinical medicine leading to advances in clinical care, medical education, and dual subspecialist training programs.^94^, ^95^, ^96^ The subspecialty harbors more questions than answers; thus, enormous research opportunities are embedded in the field.^76^ The research is expected to address gaps from basic, translational, and clinical studies to epidemiological studies and to exploit the synergistic interactions among multiple disciplines. A joint effort of multiple disciplines, including cardiology, oncology, and clinical pharmacology is to lead advances in the field of cardio‐oncology in hopes of ultimately eliminating cardiac diseases as a barrier to effective cancer therapy while providing affordable care to patients. This review focuses on the identification of the current gaps between research and clinical practice and addresses the challenges and pinpoints current insights of clinical cardiotoxicity induced by the fourth and fifth modalities of cancer treatments.

## 2 FDA‐APPROVED MTTS FOR ONCOLOGY

According to the latest reports,^97^, ^98^, ^99^, ^100^ approximately 76 targeted anticancer drugs received approval from the US FDA from 2001 to May 2017; these drugs include small molecule kinase inhibitors, other types of agents (e.g., proteasome and histone deacetylases targeting inhibitors) (Table 1), and therapeutic monoclonal antibodies (Tables 2 and 3). The clinical patterns of toxicity associated with some of these agents have been addressed or discussed in detail in other reviews.^20^, ^46^, ^47^, ^48^, ^49^ Many of the approved drugs are protein kinase inhibitors (PKIs), typically protein tyrosine kinase inhibitors (TKIs).^101^, ^102^, ^103^ Only a few inhibitors affect serine/threonine kinases (e.g., Vemurafenib and Dabrafenib),^102^ and one is a lipid kinase inhibitor (idelalisib).^104^ This distribution is in line with the fact that tyrosine phosphorylation is a unique biochemical mechanism utilized by intra‐ and intercellular communication pathways, and these kinases regulate important fundamental cellular processes, including cell proliferation, differentiation, migration, and metabolism.^103^ Protein kinases catalyze the transfer of the terminal phosphate of ATP to substrates that usually contain a serine, threonine, or tyrosine residue; this phosphate then serves as a ubiquitous mechanism for cellular signal transduction.^105^ Thus, protein kinases are ATP‐consuming enzymes with a high degree of conservation in their kinase domain,^8^, ^105^, ^106^ especially for those within the family of protein tyrosine kinases (PTKs).^2^, ^8^, ^101^ The ATP‐consuming feature is unfortunately associated with the ATP required for the vitality and viability of cardiomyocytes. Mitochondria are abundant in heart tissue, constituting approximately 45% of the myocardial volume, which is high in comparison with other tissues.^107^ The abundance is due to the high‐energy demand of the heart, which is satisfied during mitochondrial respiration; thus, more than 90% of the ATP generated by the mitochondria is utilized by cardiomyocytes for myocardial function and viability.^107^

The protein kinase family includes more than 385 serine/threonine kinases and more than 90 PTKs.^102^, ^103^, ^105^ More than 900 protein‐encoding genes with kinase activity have been confirmed.^108^, ^109^, ^110^ Out of 90 PTKs, 58 are receptors with an extracellular, transmembrane, and intracellular domain, and 32 are intracellular non‐receptors.^102^, ^111^, ^112^ Kinases comprise one of the largest classes of proteins encoded by the human genome, and their signaling molecules play an important role in regulating almost every aspect of cell function in many different tissue types and organs,^102^, ^105^, ^108^, ^109^ including the cell fate decisions of cardiac myocytes that lead to cardiac pathologies.^7^, ^113^ Thus, dysfunctional kinase activity disrupts the normal control of cellular phosphorylation signaling pathways and leads to numerous pathologies beyond cancer through pathway affiliation,^99^ including immunological, neurological, metabolic, infectious diseases, diabetes, osteoporosis, and otology.^7^, ^98^, ^101^, ^102^, ^105^, ^113^, ^114^, ^115^ Consequently, the pharmacological utility of kinase‐targeting has been expanded for kinase‐targeted therapies in a broad array of indications beyond cancers.^98^ The selectivity of drug‐kinase interactions has become a common concern in clinical pharmacology,^116^ and there is continuing debate surrounding target selection, mechanism of action, compound development prioritization, toxicity, and patient tailoring.^116^, ^117^ Kinase inhibitors also provide additional opportunities to investigate and elucidate kinase functions under various circumstances, both physiologically and pathologically.^104^ Intensive efforts in the exploration of disease targets have significantly extended the coverage of druggable targets in the human kinome from the tyrosine kinase family to several other families, such as calmodulin/calcium‐regulated kinase, glycogen synthase kinase (GSK), cGMP‐dependent protein kinase (PKG), cAMP‐dependent protein kinase (PKA), CDC‐like kinase (CLK), and protein kinase C (PKC).^98^, ^104^ In addition, targeting the ubiquitin‐proteasome pathway is an emerging concept in cancer therapy based on the hypothesis that many proteins in the proteasome are implicated in the regulation of important processes of carcinogenesis and cancer cell survival.^118^ Several proteasome‐targeting inhibitors (e.g., bortezomib,^20^, ^47^, ^119^, ^120^, ^121^, ^122^, ^123^, ^124^, ^125^ carfilzomib^20^, ^124^, ^126^, ^127^, ^128^, ^129^, ^130^) have been introduced in the clinic. A retrospective analysis of 3954 patients in phase 2/3 trials of bortezomib for the treatment of multiple myeloma reported that bortezomib‐based treatment was associated with a low incidence of cardiac events.^131^ Epigenetic cancer therapy using histone deacetylase inhibitors is also emerging,^132^, ^133^, ^134^ as epigenetic gene silencing is a hallmark of cancer cells. Two important types of epigenetic changes are DNA methylation by DNA methyltransferases (DNMTs) and histone modification by histone deacetylases (HDACs).^133^, ^134^ DNMTs and HDACs have become attractive therapeutic targets, and several HDAC inhibitors e.g., Romidepsin,^47^, ^57^, ^135^, ^136^, ^137^, ^138^, ^139^, ^140^ Belinostat,^141^, ^142^ and Panobinostat,^137^, ^140^, ^143^, ^144^ have been used in the clinic. Unfortunately, clinical cardiotoxicity has been induced by these types of drugs.^47^, ^57^, ^135^, ^136^, ^137^, ^138^, ^139^, ^140^, ^141^, ^142^, ^143^, ^144^

## 3 KINASE INHIBITORS AND CARDIAC CONCERNS

Based on kinship in structure and activity (different binding modes), kinase inhibitors can be grouped into two classes: irreversible and reversible.^102^, ^104^, ^109^, ^114^, ^116^, ^145^ Irreversible inhibitors form covalent bonds with cysteine or other nucleophilic residues in the ATP‐binding pocket.^105^, ^116^ The advantage of an irreversible inhibitor is its ability to block the active site of the kinase, which might also block mutated versions of the kinase.^101^ Theoretically, irreversible inhibitors may be superior to their reversible counterparts in several aspects. For example, they circumvent competition with high ATP concentrations in the cell and are less affected by changes in the ATP‐binding affinity, and they have prolonged pharmacologic effects, high potency, and the ability to validate pharmacological specificity through mutation of the reactive cysteine residue.^103^, ^105^ However, progress in the implementation of irreversible kinase inhibitors in the clinic remains slow because of safety and toxicity concerns,^109^, ^146^ as covalently binding kinase inhibitors require the intrinsic reactivity of cysteine‐reactive groups, which can lead to non‐selective reactions with off‐target proteins that increase toxicity.^101^, ^147^, ^148^, ^149^, ^150^

Kinase inhibitors are further categorized into four main types based on their binding mode to a target^109^, ^151^: type I inhibitors constitute the majority of ATP‐competitive inhibitors and recognize the active conformation of the kinase; type II inhibitors recognize the inactive conformation of the kinase; type III inhibitors bind next to the ATP‐binding pocket; and type IV (irreversible inhibitors) do not bind to the ATP or peptide substrate binding sites^102^, ^109^, ^116^, ^145^, ^152^ but characteristically form covalent bonds with their target enzyme.^109^, ^145^ Thus, type III and IV inhibitors are allosteric in nature.^109^, ^114^, ^150^ So‐called allosteric sites refer to locations outside of the ATP‐binding pocket, and allosteric inhibitors block protein kinase catalytic activity while having no effect on ATP binding.^109^ Allosteric inhibitors bind outside the ATP‐binding site and thus induce a conformational shift in the target enzyme to block the kinase function.^150^ Currently, the vast majority of PKIs in the clinic are type I inhibitors that target the ATP‐binding site in its active conformation (reversible ATP‐competitive inhibitors).^101^, ^102^, ^105^, ^108^, ^114^, ^150^, ^153^ With high relevance in cardiac safety liabilities, kinases have one ATP‐binding site, and thus, cardiotoxicity would theoretically be inevitable if a targeted kinase was functionally expressed in the heart (on‐target toxicity) when ATP‐competitive inhibitors are used. Even if a targeted kinase is not expressed in the heart, off‐target toxicity is also a major issue for both ATP‐competitive kinase inhibitors^154^ and non‐ATP‐competitive kinase inhibitors (allosteric inhibitors).^101^, ^147^, ^148^, ^149^, ^150^ Assuming an ATP‐competitive case, an energy system dysfunction or energy deprivation of cardiomyocytes becomes a high risk. This risk is in line with the fact that cardiac muscle tissue is highly energetic, and cardiac performance is typically reliant on aerobic metabolism as a source of ATP; impairments in this process can rapidly induce cardiac dysfunction.^155^ Pharmacologically, a theoretical solution to this problem is the development of covalent‐allosteric kinase inhibitors (type III and IV inhibitors),^156^ in which an active site‐directed moiety is tethered to another ligand that targets a location outside of the ATP‐binding cleft. In principle, allosteric inhibitors should be superior to active‐site‐directed inhibitors due to the high degree of kinase selectivity because these sites are highly divergent across the kinome and are unique to a particular kinase.^101^, ^114^, ^116^, ^151^ In contrast, the high sequence similarity in the ATP‐binding site among members of the kinase family often results in low selectivity and additional toxicities of these ATP‐competitive inhibitors. Considering the superiority of allosteric inhibitors, the development of inhibitors that target sites other than the ATP cleft has been the reality in clinic. Trametinib is the only type III inhibitor that has been approved thus far, although several promising allosteric kinase inhibitors are currently in different stages of clinical trials.^104^, ^109^ Only a few are irreversible ones (afatinib and ibrutinib)^104^ that have been approved by the FDA (Table 1). Currently, clinical cardiotoxicity induced by irreversible inhibitors, e.g., Trametinib,^20^, ^57^, ^124^, ^157^, ^158^, ^159^ Ibrutinib,^20^, ^160^, ^161^ and Afatinib^162^, ^163^ has been reported. Thus, novel mechanisms of action need to be further explored in type III and IV inhibitors.^104^ Despite the advantages of covalent‐allosteric kinase inhibitors versus orthosteric inhibitors (types I and II), whether allosteric kinase inhibitors can radically overcome the non‐selective problem (most importantly, considering clinical cardiotoxicity) while retaining their potency or effectiveness on cancer cells^104^, ^164^ in the clinical endpoint remains unclear. The ultimate answer to this question, considering cardiac safety concerns and potency on cancer cells, will derive from the accumulated clinical data in the future because the predictive values of success in the preclinical model systems are limited to the use of homogenous cell lines and immune‐compromised animals.^101^

Because protein kinases play a key role in all aspects of cancer, kinase inhibitors represent the largest family of targeted agents that have entered or are entering the clinic.^101^ However, an important concern is that only a very small percentage of kinases (approximately one‐fifth of the human kinome) have been successfully targeted clinically.^104^, ^165^ Thus, the field remains largely unexplored in the research of numerous undiscovered kinases, their respective inhibitors, and their potential cardiotoxicity/toxicities. Moreover, approximately 10–20% of the kinases are classified as pseudokinases^109^, ^166^ because of the lack of one or several of the highly conserved motifs involved in nucleotide (nt) binding or catalytic activity of protein kinases.^110^, ^165^, ^166^ Many pseudokinases in the kinome have evolved from active kinases by obtaining regulatory functions in which catalytic function is dispensable; however, a significant proportion of pseudokinases have retained their ATP‐binding ability.^166^ An important molecular property of pseudokinases is that they serve as allosteric regulators of signaling pathways.^109^ Pseudokinases also play an important regulatory role in cellular signaling, and the abnormal function of several human pseudokinases has been associated with human diseases, including cancers.^110^, ^165^, ^166^, ^167^, ^168^, ^169^, ^170^, ^171^, ^172^ Pharmacological targeting of pseudokinases may be a possible alternative option in this regard.^165^, ^173^, ^174^ For example, the receptor tyrosine kinase (RTK)‐like orphan receptors (RORs) are RTK‐like pseudokinases in this context.^175^ ROR1 is a type 1 transmembrane protein expressed on the plasma membrane and has an extracellular domain that is essential for ligand binding and signal transduction.^176^, ^177^ Interestingly, ROR1 is selectively overexpressed in many hematologic malignancies and a number of solid tumors, while it is without significant expression in normal adult tissues.^176^, ^178^, ^179^ The unique expression profile of ROR1 makes it a promising candidate for novel drug targets,^176^ assuming that this expression pattern will maximize effectiveness and minimize off‐target toxicities. A monoclonal antibody (cirmtuzumab) was developed by binding to ROR1 on tumor cells and inhibiting Wnt5a signaling, which is a pathway that is important for blocking tumor‐cell proliferation, migration, and survival.^180^ The antibody has been used in a phase I clinical trial for chronic lymphocytic leukemia.^175^, ^178^, ^179^, ^181^, ^182^

## 4 CAVEATS, CHALLENGES, AND INSIGHTS ON THE DIAGNOSIS AND THERAPY OF MTT‐INDUCED CARDIOTOXICITY

### 4.1 Caveats

An important caveat for clinicians is the fact that in many cases, the adverse cardiac events observed in the clinic were unobserved in preclinical safety evaluations or insufficiently addressed in clinical trials; these events may not even be mentioned on product labels when placed on the market.^2^, ^8^, ^58^, ^154^, ^183^ Cardiotoxicity may become apparent only in routine clinical practice.^2^ Therefore, it is important to identify new models or techniques in both preclinical and clinical settings^154^ that can better predict adverse clinical outcomes with these agents. Some studies have shown that cancer treatment‐related cardiotoxicity is the third leading cause of treatment‐associated mortality in survivors of pediatric and adolescent cancers, with recurrence and second malignancies being the two leading causes.^184^ In adult patients, cardiotoxicity is agent‐dependent, and the incidence can be as high as 50%, depending on the cardiac conditions.^185^ The incidence of treatment‐induced heart damage in pediatric cancer survivors increases over time^186^, ^187^ and can be identified many years post‐launch or even 30 years after treatment, as shown in adult survivors of childhood cancers.^186^, ^188^, ^189^, ^190^, ^191^, ^192^ A constant vigilance for cardiotoxicity is required^91^ and is especially important for pediatric cancer patients. These pediatric patients have a higher risk of developing treatment‐associated cardiotoxicity/toxicities subject to age‐associated, tissue‐specific sensitivity to cancer therapeutics^193^ and can exhibit an insidious onset (occult cardiotoxicity).^194^ Clinicians practice in a sea of uncertainty, with relatively few strategies leading to the safe prescription of anticancer drugs or use of therapeutic modalities. This uncertainty poses a threat to making life‐altering decisions based on incomplete information in real‐world practices. In addition, the so‐called “breakthrough therapy designation” (applied to many novel anticancer therapies by the FDA since 2012) 195, 196, 197, 198, 199, 200, 201, 202, 203, 204, 205, 206, 207, 208 refers to the allowance of particular drugs to have an accelerated approval timeframe in order to provide new treatment options for difficult diseases and conditions. Thus, the designation by no means guarantees an escape from cardiotoxicity of any investigative or post‐marketing drugs in clinical endpoints. Breakthrough “magic bullet” therapies have been criticized by medical professionals who emphasize the importance of evidentiary requirements for meaningful clinical data.^209^, ^210^ Beyond cancer, treatment‐related cardiotoxicity is a growing concern (not restricted to anticancer agents), and almost all therapeutic drug classes have unanticipated cardiotoxicities.^154^, ^211^ One major problem is chronically administered drugs, such as neurologic/psychiatric agents and anticancer chemotherapeutic agents, because toxicity may become evident only after long‐term accumulation of the drug or its metabolites (toxicology‐metabolic activation)^148^ in the heart.^135^, ^136^, ^193^ Cancer has been transformed from a rapidly fatal disease into a chronic condition due to advancements in detection and treatment.^212^, ^213^ As such, both short‐ and long‐term cardiotoxic effects resulting from cancer therapy are becoming more evident.^213^ The long‐term adverse effects are typically seen in adult survivors of childhood cancer, but treatment‐related cardiotoxicity can develop at any time after treatment initiation and can occur well into adulthood.^188^, ^189^, ^190^, ^191^, ^192^ Given the concern of cardiac safety liabilities in relation to non‐anticancer drugs, a particular caution should be taken when repurposing non‐anticancer drugs for novel applications in oncology.^214^, ^215^

### 4.2 Challenges, insights, and perspectives

#### 4.2.1 Current diagnostic modalities and biomarkers

Preventing cardiac damage is far more important than using a therapeutic intervention to counteract ongoing damage because in general, the heart is a terminally differentiated organ, and adult mammalian myocardium has very limited regeneration potential after injury.^45^ Thus, prompt recognition of early signs of cardiotoxicity and initiation of appropriate management prior to major clinical manifestations (i.e., the irreversible phases) is paramount for the substantial recovery of cardiac function in the hope of preventing the development of irreversible cardiotoxicity.^216^, ^217^, ^218^, ^219^ “Early detection” theoretically refers to the identification of early functional cardiotoxicity – at this stage, no morphological damage to cardiomyocytes is detectable, and the damage is usually reversible and clinically manifests as asymptomatic or subclinical cardiotoxicity.^185^, ^220^, ^221^, ^222^ Unlike functional cardiotoxicity, structural cardiotoxicity often appears in late stages and is usually irreversible, with detectable morphological and symptomatic damage.^185^, ^220^ For the detection of cardiotoxicity and the initiation of therapeutic measures in the early stages, a set of diagnostic and prognostic methods have been suggested, including clinical, imaging, serological, and molecular investigations.^198^, ^199^, ^200^, ^205^ Unfortunately, while 2D echocardiography or radionuclide angiography, ECG, and several blood markers that are commonly used in clinical practice allow the late diagnosis of cardiac dysfunction, these tests have a low sensitivity and insufficient predictive power in detecting subtle, incipient myocardial injury.^216^, ^223^, ^224^ A cardiac biopsy‐based approach is highly invasive and is not a suitable option for routinely diagnosing and monitoring cancer treatment‐related cardiotoxicity. Noninvasive or minimally invasive approaches should be considered first. Furthermore, the currently used cardiotoxicity biomarkers provide few mechanistic insights into the underlying mechanisms that lead to the identification of actionable and mechanistic biomarkers–‐understanding these mechanisms is a fundamental effort to translational medicine. Circulating biochemical markers are alternative or synergistic tools for clinical diagnosis. However, circulating biochemical markers harbor potential problems related to sensitivity and **s**pecificity with respect to systemic disease because this type of biomarker could be significantly influenced by multiple microenvironmental factors both locally and systemically. Cancer is a systemic disease with local manifestations,^225^ and cancer cachexia of multiple organs, including cardiac cachexia,^226^ is a typical reflection of its systemic nature. An ideal circulating biomarker is defined by two important characteristics: disease specificity and a linear relationship (sensitivity) between the serum/plasma concentration and disease severity.^227^ For instance, cardiac troponin (cTn), B‐type natriuretic peptide (BNP), and its N‐terminal fragment (NT‐proBNP) are the best‐studied circulating biochemical markers of cardiovascular diseases, including cardiotoxicity, and they have been used primarily in the clinic.^228^, ^229^, ^230^ However, cTn, BNP, and NT‐proBNP are elevated not only in patients with acute and chronic cardiovascular diseases but also in patients with non‐cardiovascular diseases,^227^, ^231^ including untreated cancers.^232^, ^233^, ^234^ Therefore, these biomarker values may reflect more than the disease activity to which they are applied.^228^, ^235^ Moreover, a new concern has been raised for the potential impact of the tumor itself on cardiovascular health; the underlying cancer pathophysiology may also affect cardiac biomarkers.^233^

#### 4.2.2 Application and development of advanced diagnostic modalities and biomarkers

##### 4.2.2.1 Advanced non‐nuclear molecular imaging modalities

The gap in relation to the early detection/diagnosis of cardiotoxicity should be filled by combined clinical and translational research. Advanced diagnostic imaging modalitie**s** are gaining momentum. Imaging methods have the advantage of in situ visualization of real‐time cardiac performance with dynamic profiles to assess cardiac function. Currently, advanced, non‐nuclear molecular imaging modalities in the clinic mainly include 3D echocardiography, cardiac magnetic resonance (CMR) imaging, cardiac computed tomography (CT), 3D speckle tracking echocardiography (STE), and cine images. Among these techniques, 3D echocardiography strain rate/deformation imaging, metabolic imaging, and myocardial systolic velocities seem more sensitive and should facilitate the identification of patients with more subtle measures of cardiac function.^236^, ^237^, ^238^, ^239^, ^240^ Recently, CMR imaging^241^, ^242^ and cardiac CT^243^ have been explored for their utility in evaluating cancer treatment‐induced cardiotoxicity. Cardiac CT has an excellent diagnostic performance for the detection of subclinical atherosclerosis, coronary atherosclerotic plaque, and obstructive coronary artery disease,^243^ while CMR has added value in characterizing myocardial remodeling.^241^ Conceptually, left ventricular (LV) remodeling is defined in response to myocardial injury or overload through chamber dilation and/or hypertrophy.^244^, ^245^, ^246^ Remodeling restores the contour of the chamber by removing poorly functioning areas to improve its efficiency.^247^ Classification of such states can be achieved by evaluating LV mass, LV volume, the ratio of LV mass/volume (M/V), and relative LV wall thickness (RWT).^248^, ^249^, ^250^ Thus, the spectrum of LV geometric adaptation can be measured using these geometric parameters. Ventricular hypertrophy (VH) is classified as concentric when the RWT is increased, while VH is classified as eccentric when the RWT is not increased; a third pattern, termed concentric remodeling, occurs when RWT but not ventricular mass is increased.^244^, ^248^ Analysis of cardiac function is the major focus of echocardiography, and measuring LVEF has been the clinical standard; however, LVEF has shown its limitations for early detection of myocardial dysfunction.^251^, ^252^ In the past decade, STE has become a novel clinical tool for the analysis of regional and global myocardial function, and this tool has the potential to provide a more accurate assessment of overall and regional myocardial function.^251^, ^253^ Cine images are short movies that show heart motion throughout the cardiac cycle, and the images can be very useful in studying cardiac and valvular function and the movement of blood through the heart.^254^, ^255^ As the myocardium contracts and thickens throughout the cardiac cycle, any abnormality in this wall motion indicates a problem with the myocardium, such as ischemia or infarct.^256^ Cardiac cine images have already been used to evaluate cardiotoxicity in breast cancer patients treated with trastuzumab.^257^ Although each non‐nuclear molecular imaging modality has their different strengths and weaknesses, a common feature of these modalities is the use of nonradioactive probes such as light or sound. In addition, these modalities are mainly used to detect changes in the anatomical and physiological features of cardiac events rather than function at the cellular and molecular level. In this regard, these diagnostic modalities have less value for understanding disease processes than do molecular imaging modalities (see the “Nuclear molecular imaging” section).

##### 4.2.2.2 Nuclear molecular imaging

In contrast to traditional imaging modalities, molecular imaging in the myocardium has great potential to contribute to clinical cardiovascular medicine by improving the understanding of disease processes and therapeutic mechanisms or effects,^258^ enabling the visualization and interrogation of specific biologic targets and pathways that precede or underlie changes in morphology, physiology, and function of the heart prior to the manifestation of gross anatomical features or physiological consequences.^258^, ^259^, ^260^, ^261^ Thus, the potential for molecular imaging is much greater than the detection of changes in anatomical and physiological features using non‐molecular imaging modalities, such as blood flow or contractile function.^262^, ^263^ In the preclinical setting, molecular imaging of the cardiovascular system uses multiple modalities, including optical, nuclear, MR, CT, ultrasound imaging, and fluorescence imaging.^258^, ^263^ However, clinical application in humans has been mainly restricted to the use of nuclear imaging techniques,^258^, ^262^ e.g., single photon emission computed tomography (SPECT) and positron emission tomography (PET). Nuclear molecular imaging characterizes specific disease processes (functions) in different individuals (personalized patient care) using the visualization, characterization, and measurement of biological processes at the molecular and cellular levels in humans and other living systems. Therefore, nuclear molecular imaging is different from routine X‐rays, CT, MRI, or echocardiography, which largely show how the body appears structurally rather than how it functions. However, nuclear molecular imaging uses radioactive pharmaceuticals and traces their progress through the body to “view” how the body is functioning. The application of radioactive substances in the diagnosis and treatment of disease is a unique feature for nuclear imaging. Currently, the clinical application of molecular cardiac imaging based on nuclear imaging is largely limited to the imaging of metabolism and innervation.^258^ Activation of the cardiac sympathetic nervous system is a cardinal pathophysiological abnormality associated with the failing human heart.^264^ Several SPECT and PET radiotracers can be used for imaging pre‐ and post‐synaptic function.^265^, ^266^ Among those radiotracers, the norepinephrine analog^123^ Imetaiodobenzylguanidine (123IMIBG) is FDA approved and the most commonly used agent. This agent can be used to investigate the activity of norepinephrine, which is the predominant neurotransmitter of the sympathetic nervous system.^267^, ^268^, ^269^ The discovery and application of agents such as 123IMIBG are absolutely needed in clinical nuclear imaging to provide better prognostic risk stratification, which, in heart failure, may include innervation imaging (sympathetic imaging), because this imaging modality provides more accurate information than conventional markers and mechanistic insights that drive therapeutic decisions.^258^, ^259^, ^267^ A reduced 123IMIBGmyocardial uptake or higher washout rate can predict cardiac adverse events.^123^, ^267^IMIBG imaging is more sensitive than decreased LVEF, which has been frequently used in the clinic,^268^ because a decrease in LVEF is a late manifestation of cardiotoxicity.^252^ Moreover, with the imaging modality, it is also possible to predict whether LVEF recovery will occur.^269^ Indeed, seeking early predictors of cardiotoxicity is urgently needed so that treatment can be initiated earlier or that pre‐emptive intervention can be used^270^ to prevent irreversible cardiac damage.

##### 4.2.2.3 Photoacoustic imaging

Radiotracer‐free molecular imaging modalities may serve as alternatives to nuclear molecular imaging. However, an important concern in the application of these imaging techniques in the clinic is the technical challenge of limited penetration depth in contrast to nuclear molecular imaging using radiotracers.^258^, ^262^ To overcome these technical challenges, some advances are being made to provide the required signal amplification in order to enable molecular imaging with contrast agents, e.g., new nanoparticle contrast agents coupled with MRI and ultrasound.^262^ However, the clinical safety of these contrast agents must be further evaluated in human studies.^262^ To date, no single parameter or approach can accurately predict cardiotoxicity in the clinic. Multi‐parameter test panels or modalities may be beneficial for preclinical and clinical investigations or applications because they enable the acquisition of complementary information from each panel/modality.^271^ Each imaging modality has its intrinsic advantages and disadvantages for cardiovascular events,^263^ but none of the current clinically used modalities can simultaneously image both molecular events and anatomical and physiological features. Medical diagnosis and therapeutic options benefit greatly from imaging technologies that combine molecular and microscopic parameters with clinical observations; such a combination is provided by photoacoustic imaging (also known as optoacoustic imaging).^272^, ^273^, ^274^, ^275^, ^276^ Photoacoustic imaging is a label‐free, non‐ionizing, noninvasive, high‐resolution optical imaging modality that uses optical absorption contrast and ultrasonic resolution.^277^, ^278^, ^279^, ^280^, ^281^, ^282^ This technology has a high scalability and allows imaging of biological structures, ranging from molecules, organelles, cells, and tissues to entire organs and even entire small animal bodies.^273^, ^275^, ^280^, ^283^, ^284^, ^285^, ^286^ The anatomical, functional, metabolic, and histologic properties of tissues or organs can be solely revealed by endogenous contrast (e.g., hemoglobin, lipids, melanin, and collagen), while exogenous contrast agents are only used to further increase the imaging contrast and specificity.^273^, ^283^ Various exogenous probes with high contrast have also been extensively developed, including inorganic and organic dyes,^274^ magneto‐optical and photochromic probes,^283^ nanoparticles,^282^, ^287^ and genetically encoded probes,^281^ to achieve improved resolution and sensitivity while providing multi‐parametric photoacoustic imaging.^283^ This imaging modality has proven its clinical and preclinical value in functional, structural, and molecular aspects of diseases and has been used for physiologically and pathologically imaging various organs and tissues, including breast cancer,^288^, ^289^, ^290^, ^291^, ^292^ neural tissues,^277^, ^287^, ^293^, ^294^, ^295^ fingers,^296^ sentinel lymph nodes,^277^, ^292^, ^297^ the cardiovascular system,^298^, ^299^, ^300^, ^301^, ^302^ the prostate,^303^, ^304^ skin,^305^ cancer therapy,^273^, ^274^, ^306^ muscle oxygenation,^307^ metabolic status,^274^, ^283^ eyes,^308^, ^309^ plaque pathophysiology,^310^ tumor microenvironment (pH, enzymes, radical oxidation species (ROS), and metal ions, among others),^274^ and biomaterial‐tissue interactions to assess the functions of the engineered tissue/organ constructs.^276^ Although photoacoustic imaging is conceptually different from some imaging modalities, it is complementary to many other imaging modalities. The major advantages include deep tissue penetration, good spatial and temporal resolution, a highly scalable nature, and dynamic imaging without ionizing radiation,^272^, ^275^, ^276^ thus enabling the potential acceleration of its clinical translation and application.

Economically, ultrasound‐based technologies are a valuable diagnostic tool for potential global adoption because of their affordability, availability, and portability.^273^ Currently, photoacoustic imaging has three major implementations with excellent scalability to meet the application at different levels^283^, ^284^, ^285^, ^286^, ^311^: (1) photoacoustic microscopy (PAM), (2) photoacoustic computed tomography (PAT) or multispectral optoacoustic tomography (MSOT), and (3) photoacoustic endoscopy (PAE). PAT and MSOT are seen as a future alternative to conventional scanning methods, such as MRI and CT scan.^311^, ^312^ These imaging modalities combine non‐ionizing optical and ultrasonic waves via the photoacoustic effect, which provides in vivo multiscale functional, metabolic, and molecular imaging.^313^ Photoacoustic imaging is starting to be used on patients, and the technology may revolutionize medical imaging in clinical practice.^311^, ^312^, ^313^, ^314^, ^315^ PAT/MSOT may be an optimal imaging modality for the detection of clinical and experimental cardiotoxicity. Thus far, PAT/MSOT has not been explored in clinical cardiac imaging to the best of our knowledge. However, experimental cardiac imaging using PAT/MSOT or PAM has been reported.^298^, ^302^, ^316^, ^317^, ^318^, ^319^, ^320^ The imaging modality may yield new insights into the cardiomyocytes and their life in vivo and further contribute to clinical imaging science and diagnosis.

##### 4.2.2.4 Development of novel, non‐invasive, and cost‐effective biomarkers

Multiple diagnostic tools can assess cardiac abnormalities, and cardiac biomarkers may play a complementary role to cardiac imaging in monitoring patients for cardiotoxicity.^92^ However, biomarkers that could help identify the risk for cardiotoxicity at an earlier time point require further development.^91^ Identifying risks is the first and perhaps the most important step in the risk management process. If there is a failure to identify any particular risk, then other steps in the risk management process cannot be implemented for that risk. The concept of ‘actionable’ and/or mechanistic biomarkers refers to biomarkers that are embedded or rooted in disease pathogenesis (pathogenetic pathways).^321^, ^322^ Thus, mechanistic/actionable biomarkers are more useful as predictive and/or pharmacodynamic (PD) biomarkers than as descriptive biomarkers that are byproducts of the disease process with limited value of the diagnostic and prognostic information.^321^, ^322^ Generally, those molecules involved in disease pathways most likely serve as important mechanistic/actionable biomarkers in the diagnosis and management of diseases.^322^ Thus, circulating actionable and mechanistic biomarkers of cardiotoxicity should be explored to identify cardiotoxicity risk factors.

An ideal biomarker in this context should meet the baseline criteria (superior tissue specificity, early time of release, a mechanistic association, and increased sensitivity with respect to bioanalysis).^323^ In addition, circulating biomarkers of cardiotoxicity should be able to distinguish myocardial from skeletal muscle or other tissue damage, such as cancer cachexia‐induced damage of skeletal muscle. MicroRNAs (miRNAs) are small, non‐coding RNAs of approximately 22 bp in length that post‐transcriptionally regulate gene expression by binding and inhibiting particular mRNA targets.^324^ Due to the signature profiles of their tissue specificity and disease expression, miRNAs are being extensively explored or profiled for use as disease biomarkers, including drug‐induced cardiotoxicity.^224^, ^324^ Several circulating miRNAs (miR‐1, miR‐133a, miR‐499, miR‐208, and miR‐423–5p) are promising cardiac injury biomarkers in cardiovascular diseases.^324^ miRNAs exhibit two significant characteristics: (1) they are secreted from the producing cells, and (2) they can deliver gene‐silencing signals between living cells in vitro and in vivo.^325^ The secretory mechanism of extracellular miRNAs has been explored, and the release of miRNAs into the circulation or body fluids is actively controlled through a ceramide‐dependent machinery that is associated with the secretion of small membrane vesicles called exosomes or microvesicles as a versatile communication tool.^326^ Thus, the specific miRNA profile of cardiac exosomes or microvesicles can also be utilized as a novel diagnostic tool for chemotherapy‐induced cardiotoxicity.

Regardless of the tremendous efforts made to discover novel biomarkers for clinical use, it remains unlikely that a single biomarker will be able to predict all drug‐induced cardiotoxicities; rather, an optimal panel of biomarkers that reflects multiple aspects should be developed as a multiplex test for use in the laboratory and the clinic.^211^ Further innovation is needed for the development of new noninvasive and cost‐effective diagnostic biomarkers or modalities to manage the early detection of drug‐induced myocardial injury. Compared to traditional approaches, an integrated mechanism‐informed approach (advanced molecular and cellular cardiac imaging, ultrasensitive detection of subtle electrophysiological phenotypes, circulating actionable or mechanistic biomarkers) may allow a more sensitive and specific identification of in vivo cardiotoxicity and offer a better understanding of the fundamental mechanisms for individual compounds. Identifying the early signs of cardiotoxicity would certainly be beneficial to the management of oncologic patients and become essential for identifying patients who are at risk of irreversible heart disease and for monitoring treatment outcomes. Such approaches involve the assessment of cardiac function in the broadest sense.

### 4.3 Novel therapeutic modalities in cancer versus cardiotoxicity

#### 4.3.1 Chronotherapeutic strategies using the concept of chronobiology

Biological processes and functions are organized in both space, as a physical anatomy, and time, as a biological time structure.^327^ The 24‐hr cycle in physiological processes is known as the circadian rhythm in humans and many other living organisms.^328^, ^329^ Similarly, ultradian rhythm relates to hours, minutes, or seconds; infradian rhythms refer to those spanning days or months, and longer rhythms.^330^ In general, the best‐studied chronobiologic frequency is the circadian rhythm, and cell physiology and functions are regulated along the 24‐hr time scale by the circadian timing system (CTS), which is composed of endogenous molecular clocks within each cell.^331^ The suprachiasmatic nucleus in the brain acts as the central pacemaker (a central coordination system) for the human circadian system.^331^, ^332^, ^333^, ^334^, ^335^ Chronobiology is the study of biological rhythms and the mechanisms of biological timekeeping, which is associated with the fields of medicine, pharmacology, and drug delivery.^327^ Chronotherapy addresses the use of circadian, ultradian, infradian, seasonal, or other rhythmic cycles in the application of therapy for various diseases^330^ and involves altering the timing of medication administration to improve the overall control of a disease and to minimize treatment side effects. This concept is emerging in the field of therapeutics.^336^ The initial idea in the development of chronotherapeutics was to synchronize the in vivo drug bioavailability with the rhythms of the diseases to optimize therapeutic outcomes and minimize side effects.^330^ Chronotherapeutics aim at improving treatment tolerability and efficacy through the adjustment of drug delivery to the CTS^327^, ^328^ based on the endogenous biologic rhythms.^329^ With relevance to cancer, the CTS controls several critical molecular pathways of cancer processes and therapeutic effects over the 24 hours, including drug metabolism, cell cycle, apoptosis, and DNA damage repair mechanisms.^337^ The conventional concepts of drug delivery mostly consider constant release rates to maintain drug concentrations in the human body and achieve constant drug exposure at the sites of drug effects over time to optimize treatment efficacy,^338^ regardless of the patient's physiological and biochemical conditions within the context of circadian rhythms.^329^ Drug delivery with such concepts (the homeostatic theory of drug delivery^330^) is being challenged with the advanced knowledge of the CTS; i.e., drug effects predictably vary not only as a function of dose but also as a function of administration timing.^328^, ^337^, ^339^ If a drug release profile mimics the circadian rhythms of a living system, it may improve drug efficacy and reduce the toxicity of a specific drug administration schedule.^329^ This hypothesis is in line with the main function of the CTS, which is to coordinate bodily and cellular functions, down to the main pathways that are responsible for drug pharmacokinetics (PK) and drug metabolism over 24 hours.^328^, ^340^ For example, circadian timing can improve drug tolerability and/or efficacy up to several‐fold in rodents and patients, regardless of administration routes.^328^, ^338^ The tolerability of nearly 500 medications varies by up to five‐fold according to circadian scheduling both in experimental models and patients.^340^ Thus, medication and treatments provided according to the body's circadian rhythms will result in better outcomes, and the relevance of timing may even exceed that of the dose.^341^ Improved patient outcomes on circadian‐based treatments (chronotherapy) have been demonstrated in randomized clinical trials, especially for cancer and inflammatory diseases.^340^

With advancements in the field of chronobiology, the circadian rhythms and their influence on biological systems have given rise to several mutually connected concepts beyond chronobiology, chronotherapy, and chronotherapeutics.^338^, ^342^ These concepts are extended to chronopharmacology, chronokinetics, chronodynamics, chronesthesy, chronotoxicology, and chronoprevention.^327^, ^338^, ^343^ Chronopharmacology is the study of the circadian dependencies in the PK and PD of drugs, PK‐PD relationships, and their mechanisms.^327^, ^328^, ^342^ Chronokinetics refers to dosing‐time, i.e., rhythm‐dependent differences in the absorption, distribution, metabolism, and elimination of medications.^327^ Chronodynamics refers to dosing‐time, i.e., rhythm‐dependent differences in the effects of medications.^327^, ^344^ Chronotoxicology is an aspect of chronodynamics that refers specifically to dosing‐time, i.e., rhythm‐dependent differences in the manifestation and severity of adverse effects and, thus, the intolerance of patients to medications.^327^, ^340^, ^345^ Chronesthesy refers to rhythm‐dependent differences in the sensitivity of target systems to medications that cannot be explained by corresponding administration‐time differences in PK phenomena.^327^, ^340^ The mechanisms of chronesthesy have yet to be elucidated^327^, ^340^ but may represent rhythms in receptor number and conformation, second messenger dynamics, membrane permeability, or rate‐limiting steps of metabolic pathways in drug‐targeted tissues.^327^ Chronoprevention is the timing of medications or other interventions according to biological rhythm criteria as a means of averting disease or a decline in health status.^327^ The major difference of the goals between chronoprevention and chronotherapeutics is that chronoprevention focuses on the avoidance of disease, pathology, and other deleterious phenomena that compromise heath, while chronotherapeutics are the management or reversal of existing acute or chronic medical conditions.

#### 4.3.2 Chronotherapeutic strategies in the context of precision oncology and cardiotoxicity/toxicity

Cancer is considered a chronotherapeutic disorder^332^, ^346^; thus, cancer can be a driver for system chronotherapeutics.^340^ The chronotherapy principle has been explored in the context of cancer therapies, and the results showed that circadian timing largely modifies the extent of toxicity of 40 anticancer drugs among agents in all pharmacological classes in rodents^328^ and humans.^338^, ^347^ Doxorubicin (DOX), cisplatin, 5‐fluorouracil (5‐FU) and 5‐fluoro‐2′‐deoxyuridine, irinotecan and oxaliplatin are representative anticancer agents that have been studied in the context of their circadian chronopharmacodynamics and chronotoxicologies.^327^, ^348^ The results of both laboratory animal and multicenter trials clearly show that the proper timing of cancer medications improves both patient tolerance to therapy and clinical outcomes.^327^, ^340^ For instance, the cytotoxic effect of 5‐FU was minimal for a circadian delivery peaking at 4 a.m. and maximal for a continuous infusion or a circadian pattern peaking at 4 p.m.^328^, ^349^, ^350^, ^351^ The existence of a cancer‐specific molecular clock can be used for the discovery and development of novel, therapeutic approaches to treat cancer.^352^ A pharmacological modulation of clock‐related proteins may be a suitable strategy for the identification of innovative anticancer approaches.^353^ To date, chronotherapy has been studied for some conventional anticancer agents, while data for MTT class and immunotherapies seem to be lacking. In line with the beneficial effects of chronotherapy, there is a critical and urgent need to prevent MTT and immunotherapy‐induced cardiotoxicity of cancer patients who are also using chronopharmaceutics,^338^ which may potentially improve the safety, efficacy, and patient compliance of the new generation of anticancer drugs or modalities. Additionally, CTS robustness and phase varies among cancer patients, resulting in significant variabilities in response to chronotherapy^340^ and indicating that the development of personalized chronotherapeutics is highly expected through interdisciplinary systems approaches.^337^ Personalizing cancer chronotherapeutics requires an extensive molecular knowledge of anticancer chronopharmacology and chronotoxicity, both in healthy and tumor tissues.^340^ Tumor tissues usually display a disrupted circadian organization relative to the circadian synchronization (molecular clock) in healthy tissues,^327^, ^348^ although the molecular basis of this difference remains unclear.^340^ Moreover, circadian disruption has been observed in up to 50% of patients with metastatic cancer, and such disruption was associated with poor outcomes.^337^ Conceivably, the differential molecular clock between cancer and normal tissues can be exploited in treatment timing to specifically shield healthy cells while targeting cancer cells.^340^, ^354^, ^355^ Indeed, a combination of mathematical, statistical, technological, experimental, and clinical expertise is now shaping the development of dedicated delivery algorithms that enable treatment individualization (patient‐tailored chronotherapies).^330^

The concept of chronotherapy offers further potential for improving current cancer treatments and optimizing the development of new anticancer or supportive agents.^328^ Clock modulators or mediators are often considered natural candidates for a chronotherapeutic approach.^352^ One important mediator of circadian activity is the hormone melatonin (MEL), which peaks at the end of dark periods in both diurnal and nocturnal mammals.^356^ Therefore, MEL has been used to mimic the dark period in humans as a means of treating sleep disorders and jetlag.^357^. The determination of the circadian phase in individual patients through monitoring relevant CTS biomarkers could drive personalized chronotherapy and improve treatment effects,^337^ with particular significance in cancer therapies.^358^ CTS biomarkers mainly involve circulating MEL, cortisol, body temperature, and rest–activity rhythm.^331^ For example, DOX should be given mid‐morning when circulating MEL levels are low to further reduce cardiotoxicity from DOX treatment.^359^ Mitochondria are a common target for both MEL and many anticancer agents.^107^ The heart is particularly susceptible to oxidative damage induced by anticancer agents because it is abundant in mitochondria,^107^ which are both sources and targets for ROS.^360^ Furthermore, the heart has an elevated rate of oxygen consumption and limited antioxidant defense systems when compared with other tissues. Thus abnormalities in mitochondrial functions may be the primary causative factors in the pathogenesis of anticancer drug‐induced cardiotoxicity.^107^, ^361^

In view of the abovementioned information, the general clinical approach to attenuate anticancer drug‐induced cardiotoxicity is to utilize natural antioxidants such as MEL. The dark hormone holds amphiphilic properties. The term “amphiphilic properties” refers to a chemical compound possessing both hydrophilic and lipophilic properties. MEL has shed light on this therapeutic avenue with being dually oncostatic and cardioprotective.^107^ Another appealing and unique property of MEL, which other antioxidants do not possess, is that its metabolites also exhibit antioxidant activity by scavenging ROS and reactive nitrogen species (RNS).^107^ In addition, MEL concentrations in subcellular compartments (cell membrane, cytosol, nucleus, and mitochondria) fluctuate independently of the circadian rhythm during a 24‐hr period.^107^ From a therapeutic perspective, clinicians should bear in mind that the subcellular levels of MEL are controlled by regulatory mechanisms; therefore, MEL has low toxicity when administered at high doses.^362^ MEL is the first known natural antioxidant molecule that may have both curative and palliative actions in the treatment of human neoplasms.^363^ To date, studies regarding the direct effect of MEL on the mitochondrial respiratory chain and complexes in relation to anticancer agent‐induced cardiotoxicity, including MTT, seem to be lacking.^107^, ^364^ This crosstalk between MEL and the mitochondria is quite important as it can enable to overcome these pharmacological hurdles for greater clinical impact. Because almost all clinically used antitumor drugs exhibit toxic side effects that affect heart function,^365^ the discovery of natural phytochemicals as chronopharmaceuticals for clinical use becomes more and more important. Aside from MEL, other natural phytochemicals such as coenzyme Q10, L‐carnitine, resveratrol, curcumin, and ginkgo biloba also counteract the cardiotoxic side effects of cancer chemotherapy.^365^ Thus, co‐administration of these natural cardioprotective agents seems promising to improve the clinical outcomes of cardiotoxicity.

In light of emerging chronotherapeutic approaches,^366^ various chronoprogrammable drug delivery systems have been developed.^329^, ^339^, ^367^, ^368^, ^369^, ^370^, ^371^, ^372^, ^373^, ^374^, ^375^, ^376^, ^377^, ^378^, ^379^, ^380^, ^381^ These approaches include chronomodulating infusion pumps, controlled‐release microchip strategies, floating pulsatile systems, nanotechnology, press‐coating approaches, micro‐electrochemical systems, osmotic pressure, microchips, liposomes, thermosensitive hydrogels, micro‐ and nanocarriers, and microparticle‐based systems. The development of programmable time pumps has enabled the safe and highly effective delivery of combination chronotherapy protocols.^337^ Unfortunately, no current drug delivery system can satisfy all the requirements of chronotherapeutics.^330^ From the applied and clinical perspectives, one of the most important issues is to consider the ease of manufacturing and cost‐effectiveness during the selection of the appropriate chronopharmaceutical technology.

## 5 RESEARCH GAPS, CHALLENGES, PERSPECTIVES, AND OPPORTUNITIES TO REDUCE MTT‐INDUCED CARDIOTOXICITY

### 5.1 The gaps in cross‐disciplinary research

The lack of a mechanism‐based curative treatment of cardiotoxicity for many compounds is a treatment concern in the clinic, and only general cardioprotective care or discontinuing cancer therapy (premature discontinuation of chemotherapy) is currently applied to the patients. Each molecular drug or therapeutic modality may have a unique biological mechanism of action because each drug shows a unique toxicity profile regardless of whether the drugs are in the same class and exhibit structural similarity.^382^ This feature is also supported by the observation that transcriptome‐wide response profiles in cardiomyocytes using a cohort of KIs showed a limited overlap.^383^ Therefore, general cardioprotective strategies (ACE inhibitors, beta‐blockers, digitalis, etc.) may not work effectively. Disease‐specific mechanisms must be addressed in preclinical research (basic and translational) for a mechanistic understanding of the cardiotoxicity induced by novel anticancer therapies. Drug‐induced cardiac dysfunction and cardiomyopathies reflect serious, clinically adverse effects of drug actions, and they can be used in experimental models to study the pathogenic mechanisms of these cardiac disorders, offering the advantage of precise control of the onset time, and can often be studied in a longitudinal fashion.^384^ The gaps currently observed between clinical observations and pharmacological innovation might be reduced by a better application of the concept of reverse translational research with a bidirectional research paradigm ‐ from practice (bedside) to research (benchside) ‐ and a back flow of information. This research paradigm aims to transfer clinical insights or observations into hypotheses that can be investigated in the basic research laboratory and that can subsequently inform clinical practice. With such a bidirectional research paradigm, clinical cardiotoxicity becomes an important source of observations and ideas to feed into fundamental research for further mechanistic insights. In turn, these new mechanistic insights allow proposing novel concepts regarding these drugs and accelerating clinical advances in knowledge to further bridge knowledge gaps. Such a mechanistic approach should yield disease‐onset, mechanism‐based biomarkers that identify the initial disease stages. These markers are valuable tools for developing strategies to prevent the progression of diseases that can be translated into clinic for diagnosis and to measure disease progress and/or the effects of treatment. In line with the expectations, using various modeling systems (in vitro, in vivo, computational biology or their combination) to explore the underlying mechanisms that drive the cardiotoxicity is a high‐priority research area in preclinical research.

The development of new PD, prognostic, and surrogate biomarkers not only enhances the efficiency of cancer treatment (e.g., targeting neoantigens) but also mitigates the risk of cardiotoxicity. Thus, this development is urgently needed in this era of precision oncology.^93^ According to the hierarchical tree of various sources of biomarkers in nature (genes and genetic variants, RNA, microRNA, proteins, and metabolites), some caveats should be kept in mind: (1) each category of markers is suited to a specific purpose, (2) caution should be taken regarding cross‐species translatability, and (3) biomarker values fluctuate in response to the pathophysiology of the organism over time. Taken together, genomic and transcriptomic markers can provide information about a person's risk of developing a specific disorder or how a patient may respond to treatment. Proteins and metabolites are more dynamic in a disease course and thus carry more diagnostic information than DNA or RNA. It is important to note that DNA, RNA, and proteins are more species‐dependent; thus, these biomarkers should be best identified and characterized using human materials. In contrast, small molecules or metabolites are less species‐dependent,^385^, ^386^ and miRNAs are also well conserved among species, ranging from worms to humans; 324, 387 therefore, many of these markers from animal studies can be translated into clinical practice.^324^, ^388^ Metabolic biomarkers are subtly sensitive indicators of health status that provide early profiles on drug efficacy, toxicity, and mechanism.^45^, ^389^ miRNAs are sensitive and specific toxicity reporters as well.^224^, ^390^ Considering the sensitivity and interspecies translatability, identifying metabolic biomarkers, especially metabolic imaging biomarkers and/or circulating/tissue miRNA markers, should be prioritized in preclinical studies using model organisms, allowing a direct comparison of animal models with human studies. Altered ionic homeostasis is considered the foremost change in the early phase of responses of myocardium to environmental toxicants or drugs.^45^ Preclinical research on the molecular basis of cardiac ion channels in relation to regulation and drug sensitivity of the cardiac ionic currents should be performed.

### 5.2 Modeling systems for clinical cardiotoxicity

#### 5.2.1 Overview of living modeling systems

The next step is to select a proper modeling system for predicting clinical cardiotoxicity as precisely as possible. A proper model should encompass adequate sensitivity, predictability (similarity to human myocardial biology), measurable functional parameters of cardiac dynamics/performance, real‐time data acquisition over time, and high throughput, all of which should be considered collectively. For example, in vitro assays that use primary animal cardiomyocytes, human‐induced pluripotent stem cell (hiPSC)‐derived cardiomyocytes, hiPSC‐derived endothelial cells, and hiPSC‐derived cardiac fibroblasts are primarily designed as electrophysiological models to assess the interaction of drug candidates with the main ion channels that are involved in maintaining the cardiac action potential (electrophysiology),^391^, ^392^ contractility,^392^ kinase phosphorylation profiling^392^ and more. Complementary to these in vitro assays, mathematical or computational approaches can model the propagation of the action potential and ion concentration dynamics of the heart.^393^ Recently, the xCELLigence RTCA Cardio System was developed to monitor cardiac contractility and arrhythmogenic properties in vitro based on impedance measurement. This technology can be used for a high‐throughput screening of functional cardiotoxicity.^391^ However, many in vitro models may lack or incompletely recapitulate important aspects of human biology in vivo,^8^, ^394^ allowing certain toxicities to escape detection. Cardiac performance is the result of an entire functioning organ, including electrophysiological function (rhythms or heartbeats), pump function (cardiac output, stroke volume, stroke work, ejection fraction) and mechanical muscle function (force velocity curve, maximum velocity of shortening (Vmax), LVEF, ventricular contractility (dP/dt)). Although ex vivo experiments using isolated hearts from live animals (e.g., the Langendorff heart assay) can be used to assess cardiac performance, these experiments present technical challenges because they are labor intensive and not amenable to a high‐throughput analysis. However, addressing in vivo cardiac effects of MTT necessitates a suitable animal model. Thus, it is important to select the most appropriate animal model for mechanistic studies and/or as a predictive preclinical model.

#### 5.2.2 Rodent model

In biomedical sciences, *Mus musculus* is the most commonly used species for animal models due to its genetic, physiological, and anatomical similarities to the human system in general. However, the expenses associated with the use of this species limit its application in large‐scale molecular and/or therapeutic screening or modeling. To date, using a mouse model solely for drug screening (i.e., a single factor experimental study) is very rare, while multi‐factorial experiments have often been performed, for example, simultaneous evaluation of antitumor efficacy and cardiotoxicity of cancer drugs,^395^, ^396^, ^397^, ^398^, ^399^, ^400^, ^401^, ^402^, ^403^, ^404^, ^405^, ^406^ and/or studies of cardioprotective strategies after using cancer drugs.^407^, ^408^, ^409^, ^410^, ^411^, ^412^ More importantly, many drugs, including oncologic pharmaceuticals, can often lead to cardiotoxic electrophysiological effects (e.g., QT prolongation, atrioventricular conduction blocks, and ventricular arrhythmias including torsades do pointes); thus, adverse electrophysiological effects represent important phenotypes of drug‐induced cardiotoxicity.^75^, ^391^, ^413^ In this regard, the intrinsic species‐specific differences of cardiac electrophysiology require attention during model selection. The cardiac electrophysiological properties in mice are significantly different from their human counterpart.^414^ Furthermore, mice are nocturnal animals; therefore, this CTS of this species is mismatched with the human counterpart from the perspective of chronobiology and chronopharmacology. These practical concerns may lead one to consider mice as a second choice model organism for studying drug‐induced cardiotoxicity.

#### 5.2.3 Zebrafish model

Zebrafish have filled a niche in the phylogenetic gap between invertebrates and mammals as one of the most successful vertebrate models for studying human physiology and diseases.^415^ Approximately 70% of human genes have at least one obvious zebrafish orthologue and mutations in homologous genes lead to similar phenotypes.^416^ The genome of zebrafish is well conserved, and its physiology shows good resemblance to mammals in general. Additionally, most zebrafish organs perform the same functions as their human counterparts.^417^ Regarding the cardiovascular system, zebrafish stand out for their highly conserved integrative physiology of the cardiovascular system and a pharmacological response similar to that of human beings.^417^, ^418^ The key morphological, functional, mechanobiological, electrophysiological, metabolic, and molecular profiles, as well as many cardiac events, overlap with those in humans,^418^, ^419^, ^420^, ^421^, ^422^ including the kinome profiles, where most kinase inhibitors interact with the kinases.^423^, ^424^ Moreover, the cardiac electrophysiological properties of the hearts of zebrafish larvae and adults resemble those of humans in many aspects.^413^, ^425^, ^426^, ^427^, ^428^ It is of particular importance to evaluate the pharmacological response of cardiac function in an animal model relevant to the human heart for an appropriate assessment of the safety (drug‐induced cardiotoxicity) and efficacy of drugs that target cardiovascular diseases (cardiopharmacology). Many human cardiovascular drugs have identical effects on zebrafish physiology, and numerous human cardiovascular disorders have been recapitulated in zebrafish genetic models.^417^, ^429^ Importantly, many drugs that cause QT prolongation in humans consistently cause bradycardia and AV block in zebrafish,^413^ suggesting that zebrafish are a rational, predictive model for cardiac safety liability of chemicals/drugs and also a valued, mechanistic model of human cardiovascular diseases. The overall physiology, genetics, and cardiovascular pharmacology similarities strongly support the use of zebrafish as the most reasonable approach for modeling human cardiovascular diseases or events in vivo,^420^, ^428^, ^430^, ^431^, ^432^, ^433^ monitoring drug‐induced in vivo cardiotoxicity,^413^, ^417^, ^418^, ^434^, ^435^, ^436^ and in vivo cardiovascular drug discovery.^429^ Furthermore, zebrafish are a diurnal vertebrate;^437^ thus, the zebrafish CTS is expected to be compatible with the human counterpart for studies of chronobiology and chronopharmacology.^437^, ^438^, ^439^, ^440^, ^441^, ^442^, ^443^, ^444^, ^445^, ^446^, ^447^, ^448^, ^449^, ^450^, ^451^, ^452^, ^453^, ^454^ Recently, zebrafish have been increasingly recognized as a model for circadian rhythm disorder.^455^ Lastly, the general advantages of the zebrafish model,^456^ e.g., convenience of drug delivery, rapid development, ease of genetic manipulation, low cost of maintenance relative to other model animals, high fecundity, transparency, small size, and the availability of various transgenic lines, make this animal model an almost ideal complement to rodent‐based biomedical research. This organism has been considered the “new mice” to replace the so‐called “higher vertebrates” (e.g., mice, rats) according to the 3R (replacement, reduction, and refinement) perspective for animals used for experimentation.^456^, ^457^, ^458^ Research in exploring the potential use of zebrafish as a mechanistic model organism to study cancer treatment‐induced cardiomyopathy and myocardial dysfunction or to exploit the predictability of clinical cardiotoxicity, particularly for MTT, should be highly promoted. To date, extensive studies of the mechanisms involved in relation to MTT have not yet been undertaken, and published data in the preclinical setting using zebrafish remain highly limited.^434^ A research program is being started in our laboratory using zebrafish as a predictive and mechanistic model to address clinical cardiotoxicity induced by MTT to identify potential mechanistic biomarkers.

Regardless of the aforementioned merits, several important caveats in modeling kinase inhibitors on a zebrafish model should be considered: (1) Confounding effects: transgenic fluorescent animals are genetically modified, and the confounding effects of an inbred strain background may harbor compensatory mechanisms that can alter biological events.^459^ Thus, any data obtained from transgenic lines must be further confirmed by wild‐type fish lines. (2) Cardiac regeneration: the zebrafish heart maintains the ability to regenerate throughout adulthood with scar‐free regeneration of a damaged heart. In contrast, the adult mammalian heart exhibits a limited signaling powerhouse and cell reservoir for regeneration.^460^, ^461^ The limited cardiac regenerative capacity in humans is witnessed by scar formation following myocardial infarction. (3) The human kinome shares many catalytic domains of zebrafish homologs,^423^, ^462^ which is important for tyrosine kinases.^462^ However, small changes in the sequence of amino acids in a protein may reduce or enhance certain inhibitor interactions, consequently leading to under‐ or over‐estimation of toxicity.^423^ To extend zebrafish utility as a functional model organism for toxicity and efficacy in studying kinase inhibitors, more knowledge of these relationships may be required. (4) Phenylthiourea (PTU) is a well‐known inhibitor of tyrosinase^463^ and is widely used in zebrafish research to suppress pigmentation in the developing embryos/larvae of small animals.^464^ In modeling the toxicity of kinase inhibitors on zebrafish, PTU is not recommended to avoid potential synergistic effects from PTU and a test drug. (5) Pseudokinases are generally less conserved between zebrafish and humans.^423^ Thus, it is unsuitable to model pseudokinases in a zebrafish model. (6) The drug exposure method for zebrafish is mainly bathing, and caution should be taken with insoluble drugs and drug metabolism.^465^, ^466^ Therapeutic antibodies are large therapeutic molecules that differ from small molecule agents in absorption, distribution, and elimination properties between the two classes of drugs in a systematic manner.^467^ (1) Absorption: the absorption of small and large molecules differs with respect to their common extravascular routes of delivery (oral for small molecule agents versus injection for antibodies). (2) Elimination: small molecule agents are commonly distributed into the tissues but are eliminated primarily by liver metabolism. Meanwhile, catabolism and target‐mediated drug disposition are unique features of antibody distribution and elimination.^468^ The above knowledge must be considered when testing humanized antibodies in animals.

### 5.3 Challenges, perspectives, and opportunities

#### 5.3.1 Kinase inhibitors versus “precision oncology” on efficacy and cardiotoxicity/toxicity

Introduction of the “MTT for cancer” concept in the past 15 years has led to optimistic expectations that we can precisely target the molecular underpinnings or molecular drivers of a patient's disease, and this concept is hailed as a revolutionizing approach in the treatment of cancer.^469^ Most impressively, the concept has served as the foundation for personalized medicine or precision oncology/medicine to guide the selection of treatments in the clinic.^470^ Precision oncology aims to deliver the right therapy to the right patient at the right time through promising identification of genetic alterations in human cancer and either the signaling pathways or specific biological processes that are essential for the development and progression of tumors, followed by therapy targeted to a patient's unique genetic or other relevant characteristics.^471^, ^472^, ^473^, ^474^, ^475^, ^476^, ^477^, ^478^, ^479^ Many kinase inhibitors that target oncogenic mutations in protein kinases have been developed under such theoretical expectations and perspectives.^115^, ^480^ Consequently, most kinase inhibitors that are currently developed and clinically used largely focus on anticancer effects. In real‐world practice, an individually tailored cancer treatment is determined or guided by specific molecular biomarkers based on a genetic understanding of their diseases (biomarker‐informed treatment).^2^, ^481^ These biomarkers are somatic genomic alterations (mutated, amplified, or overexpressed), often called “driver mutations,” and are considered responsible for the initiation and progression of cancer. Thus, these “driver mutations” are optimal biomarkers for selecting patients with targeted therapies.^481^ Because mutations and dysregulation of protein kinases play causal roles in cancers, kinase inhibitors have become the largest class of new anticancer drugs, serving as the cornerstone of the development of molecularly targeted therapeutics to date,^2^, ^109^, ^472^ although the effectiveness/potency and potential cardiotoxicity/toxicity of those very recently approved therapeutics remains unclear.^2^ In light of the promise of precision oncology and based on the concept of MTT over the past 15 years, many concerns and challenges have been raised in the real world, and the medical, pharmacological, and research communities must face those challenges. First, clinically, intrinsic (primary), and acquired (secondary) resistance to targeted agents has emerged as a primary challenge.^471^, ^482^, ^483^ This resistance is typically reflected by the limited effectiveness of targeted therapies on long‐term clinical benefits. Almost all patients acquire resistance in less than two years from therapy initiation, and a small subset of patients (10–20%) simply fail to respond at all, demonstrating intrinsic (primary) resistance.^116^, ^471^, ^482^, ^483^ This resistance is because of alternative oncogenic pathways taking over and/or other mechanisms (e.g., bypass pathways), leading to the occurrence of drug‐resistant variants.^101^, ^471^, ^482^, ^484^, ^485^, ^486^, ^487^, ^488^, ^489^ Cancer cells may become drug resistant much more rapidly than would be predicted from the rates of conventional mutation^116^ by differential utilization of genomic and epigenetic strategies at various levels through point mutations, deletions, translocations, amplifications, altered microRNA levels, and epigenetic anomalies (epimutations).^134^, ^490^, ^491^ Therefore, irreversible inhibitors may also need to be combined with other targeted agents and/or chemotherapy to address drug resistance.^101^ In addition, secondary mutations in the ATP‐binding site, such as threonine to methionine at position 790, serve as a mechanism of resistance to ATP‐competitive kinase inhibitors.^492^ The complex and adaptive nature of most tumors frustrates the optimistic predictions of MTT for cancer.^493^ Second, most of these PKIs prolong survival in cancer patients only weeks or months longer than standard cytotoxic therapies.^109^ In other words, generally targeted therapies by inhibiting protein kinases or other molecular events are usually not curative, even when combined with other chemotherapy or radiotherapy.^101^, ^494^ Third, the number of patients who gained clinical benefits from genomic profiling or the analysis of their tumor genomes followed using MTTs against those “driver” mutations is currently quite small,^470^, ^495^ and responsive patients may be designated “exceptional responders” for largely unexplored reasons.^494^, ^496^, ^497^, ^498^, ^499^, ^500^ To this end, inhibiting tumor growth through targeting defects in cancer genetics does not seem working meaningfully, while life‐threatening complications such as cardiotoxicity are unavoidable, uncontrollable, and unpredictable. Fourth, the majority of cardiotoxicities induced by MTTs are unexpectedly associated with the second‐ and third‐generation kinase inhibitors;^501^ a new face on an old problem. Fifth, the paradigm of precision oncology remains a theoretical promise or hypothesis rather than a real‐time reality in many areas of clinical oncology.^479^, ^495^, ^502^

#### 5.3.2 Kinase inhibitors versus cancer driver or actionable mutations on efficacy and cardiotoxicity/toxicity

Kinase inhibitors that are designed to target cancer “driver” mutations, which are identified by cancer genetic profiling, are being challenged for their clinical endpoint—i.e., the clinical beneficial effects from targeted agents are not generalizable to a large patient population.^101^, ^109^, ^471^, ^482^, ^483^, ^494^ First, biologically, the “driver”‐centric paradigm on tumorigenesis is being challenged not only by the clinical reality but also by a novel paradigm.^503^, ^504^, ^505^, ^506^ The novel paradigm envisions that those traditionally considered “passenger” mutations (i.e., those mutations with no roles in carcinogenesis) affect the course of cancer progression.^504^, ^505^, ^506^ Further, cancer evolution and progression are a balance of “driver” and “passenger” mutations, rather than being solely determined by “driver” mutations.^505^, ^506^ In line with the novel paradigm, the role of a targeted therapeutic approach is to interfere with an existing balance of “driver” and “passenger” mutations of cancer and then to develop a new interaction network to build a new balance of “drivers” and “passengers”. Such an iterative cycle can occur through an entire treatment course with different kinase inhibitors or combined with other categories of anticancer drugs in real time. As observed in the clinic, most tumors can escape from the inhibition of any single kinase, as manifested as a partial and/or non‐durable response with these therapeutics, and multiple target inhibitors are necessary to sort the effects.^101^, ^153^, ^483^, ^507^, ^508^ The balance between “drivers” and “passengers” reflects the clinical reality, and this novel paradigm may encourage the clinical and pharmaceutical communities to rethink and refine the current concept of MTT under the umbrella of precision oncology, with respect to the pharmaceutical innovation against the effects on the clinical endpoint. Second, the emerging concept of “actionable mutations”, defined as those mutations that are potentially responsive to a targeted therapy (actionability),^509^, ^510^ remains a huge challenge with respect to the identification of such targetable mutations.^494^, ^509^ From the perspective of onco‐pharmacology, the most significant success has been achieved in oncology by targeting mutationally activated “oncogenic” driver kinases, including Bcr‐Abl, EGFR, c‐Kit, PDGFR, ALK, and b‐RAF (Tables 1 and 2).^105^, ^469^ To date, only a small subset of the human kinome has been studied,^104^ and many potential candidate kinases for cancer therapies remain unexplored.^469^, ^511^ Consequently, the cardiac safety liabilities of those potential future kinase inhibitors in the clinical endpoint are unknown. Third, with more than 518 protein kinases in the human kinome,^108^ including the cardiac kinome,^512^ it remains largely ambiguous in relation to the target specificity or selectivity of individual kinases and their biological roles in the cells or tissue‐specific contexts, and most of the identified kinases remain largely uncharacterized or unexplored for their functions and tissue expressions,^513^ including the majority of kinases expressed in the heart.^514^ Many molecular mechanisms relevant to cardiac disease are also relevant to tumor biology, suggesting that cancer and cardiovascular disease have a shared biological mechanism.^515^ Indeed, numerous overlapping signaling pathways that drive tumorigenesis are also essential for normal cardiac function.^3^, ^7^, ^512^, ^516^ Unfortunately, these molecular signaling pathways are often targeted in molecularly targeted cancer therapies.^8^, ^517^ Fourth, promiscuous targeting is a unique feature of ATP‐competitive kinase inhibitors, and the potential is greater than almost any other type of drugs regardless of multi‐targeted or highly specific targeted kinase inhibitors.^50^, ^153^, ^518^ Multi‐targeted agents are frequently more efficacious than selective agents,^116^ and partial inhibition of a small number of targets can be more efficient than the complete inhibition of a single target.^519^ However, a major concern using combination approaches is the possibility of more uncontrollable and unpredictable toxicities on multiple organs/tissues than that observed with single target approaches. Therefore, target selectivity/specificity represents the biggest challenge for drug design and the development of kinase inhibitors^116^, ^483^ because most inhibitors interact with the highly conserved ATP‐binding sites of kinases. Additionally, developing pharmacological agents that target only one of the more than 500 kinases present in humans remains a formidable challenge.^164^

#### 5.3.3 Effects of kinase inhibitors on the immune system and concerns for autoimmune cardiovascular diseases

Kinases play pivotal roles in tumor immunity and tumor immune evasion; thus, they could serve as relevant therapeutic intervention points far beyond various antibody‐based therapies.^520^ However, it is important that a kinase inhibitor does not co‐target components of the immune system that are essential for mounting an immune response.^101^ Unfortunately, some drugs may bind directly and reversibly to immune receptors, e.g., major histocompatibility complex or TCR, thereby stimulating the cells in a manner similar to a pharmacological activation of other receptors.^521^ This concept has been termed “pharmacological interaction with immune receptors” or the “p‐i concept”.^521^, ^522^, ^523^ In line with the importance of kinases in the regulation of an immune response as an intrinsic defense mechanism,^524^ many oncogenic signaling pathways in tumor cells, such as MAPK or PI3K/AKT/mTOR, are also critical in the regulation of immune cells, and those oncogenic signaling pathways are often targeted by anticancer kinase inhibitors.^525^ Thus, many targeted agents might have “off‐target” effects (either beneficial or detrimental) on immune cells beyond their intended effects on the respective signaling pathways in cancer cells.^526^, ^527^ Immunomodulatory effects of kinase inhibitors can act individually on DCs, effector T cells, and immunosuppressive cells or their combination.^528^ Indeed, such effects are induced by some FDA‐approved kinase inhibitors, e.g., temsirolimus, sorafenib, sunitinib, imatinib, and dasatinib.^101^, ^525^, ^529^ The secondary pharmacological interaction of kinase inhibitors with the immune system have yet to be studied in detail in cancer patients,^525^ and the exact in vivo mechanisms remain to be further clarified in human studies. This crosstalk may open new possibilities for using either a stimulatory or inhibitory function of the immune system for defined targeting.^521^ The consequences of these kinase inhibitors on the functionality of immune effector cells alter immune cell infiltration (immune subset conditioning), increase the frequency and function of effector immune elements, and decrease the number and function of immune suppressor cells.^528^, ^529^ Although the regulation of immune cell signaling with kinase inhibitors has produced robust evidence, whether autoimmune cardiovascular diseases can be induced by the pathway when patients are treated with kinase inhibitors remains unknown. Further studies are warranted to determine the causal direction of this relationship.

#### 5.3.4 Targeting non‐coding cancer drivers by kinase inhibitors and concerns of cardiac safety liabilities

Cancer may arise from the accumulation of multiple driver mutations.^530^ However, cancers harbor a large number of molecular alterations, and targeting one or some of the many molecular alterations to achieve a clinically significant, sustained effect might not be realistic for most tumors.^479^ In addition, the overwhelming majority of oncogenic variants, both somatic and germline, occur in non‐coding portions of the genome^531^ as opposed to protein‐coding regions, which have been incorporated into the paradigm of precision oncology in the clinic. The concept of MTT developed to target protein‐coding cancer drivers is being challenged by the emerging paradigm of non‐coding cancer drivers.^531^, ^532^, ^533^ The non‐protein‐coding cancer genome remains widely unexplored,^532^ but this field will have a profound impact on many disciplines beyond cancer research. For example, such genetic events noticed in cancer are also observed in autoimmune disorders, in which approximately 90% of the causal variants are non‐coding, and most map to immune cell enhancer regions.^534^ Given the central role in cellular signaling of kinases in the heart and many other organs aside from cancers, accumulating pharmacological and pathological evidence has revealed that kinases are also promising drug targets for numerous non‐oncological indications.^101^, ^535^, ^536^, ^537^, ^538^, ^539^, ^540^ The successful approval of tofacitinib for the treatment of arthritis is a typical example,^536^ although the exact mode of action of tofacitinib in the setting of autoimmune disease has yet to be unraveled.^537^ The importance of cardiac safety liabilities with kinase inhibitors goes beyond cancer treatment. Insights gained from the clinical endpoints of oncology show that an optimization of the therapeutic equation (efficacy vs. toxicity) appears to be very hard to achieve in many cases with kinase inhibitors.^116^ It is highly important to use robust systemic approaches,^154^, ^541^, ^542^ including kinase panels^543^ and chemical proteomics,^544^ for understanding chemical interactions with biological systems, characterizing drug‐induced molecular changes in affected cells and tissues of interest, and permitting kinome‐wide analysis of candidate molecules.^116^ These strategies may allow researchers to start with these theoretical approaches to initially identify virtual cardiotoxicity by focusing on pathways that are common to all or many cardiotoxicities.^154^, ^545^ Then, a multitude of data will help reach a better characterization of “specific” tumor or disease signaling pathways that are unique to cancer cells or diseases and do not significantly affect normal cells such as heart muscle.

## 6 CARDIOTOXICITY INDUCED BY ICIS AND T‐CELL THERAPY

Immune therapies represent an advance in the fight against cancer. In a broad sense, this field encompasses a number of treatment approaches that utilize distinct components of the immune system in the fight against cancer.^44^, ^546^ The main forms of immunotherapy strategies that are used or in active clinical development today include ICIs, therapeutic monoclonal antibodies, cancer treatment vaccines, immune system modulators, and immune cell therapy (including different forms of adoptive cell transfer (ACT), such as tumor‐infiltrating lymphocytes (TILs) and CART). Currently at the forefront of immunotherapy are ICIs and CART. Here, we focus on ICIs and CART cell therapy and their potential to induce cardiotoxicity. To date, clinical cardiotoxicity induced by ICIs and CART seems to be less frequently reported compared with MTT and other categories of anticancer drugs. The clinical data remain limited because the approved ICIs are still limited, and CART has not yet been approved. Thus, serious side effects such as cardiotoxicity are still being documented. Nevertheless, clinical cardiotoxicity induced by ICIs^12^, ^13^, ^14^, ^15^, ^16^, ^17^, ^18^, ^19^, ^20^, ^21^, ^22^, ^23^, ^24^, ^25^, ^26^, ^27^, ^28^, ^546^, ^547^, ^548^, ^549^, ^550^ and CART^30^, ^31^, ^32^, ^33^, ^34^, ^35^, ^36^, ^37^, ^38^, ^39^, ^40^, ^41^, ^42^, ^43^, ^44^ have been reported (Table 3).

In the physiological situation, immune checkpoint proteins limit the strength and duration of immune responses and normally act as a type of “off switch” that helps keep the T cells from attacking normal cells in the body,^551^ which in turn reduces autoimmunity and promotes self‐tolerance.^552^, ^553^, ^554^ Recently approved ICIs that target receptors that are involved in the immune escape of cancer cells, such as cytotoxic T lymphocyte‐associated antigen‐4 (CTLA‐4), programmed cell death protein‐1 (PD‐1), and programmed cell death protein ligand‐1 (PD‐L1), are increasingly being used for therapeutic benefit in a number of cancers.^546^ Thus far, the development of ICIs has focused on these major targets.^14^ These therapeutic monoclonal antibodies interact with specific co‐stimulatory or co‐inhibitory molecules that are expressed on the surface of activated T cells and strengthen the immune response against cancer cells and minimize tumor evasion from host immunity.^555^, ^556^ ICIs work by blocking inhibitory pathways of T‐cell activation, leading to an immune response directed against tumors; thus, this activation represents a nonspecific immunologic activation that can lead to immune‐related adverse events (IRAEs).^557^ To date, FDA‐approved ICIs 26, 27, 547, 548, 550, 558 include Ipilimumab (CTLA‐4), Pembrolizumab (PD‐1), Nivolumab (PD‐1), Atezolizumab (PD‐L1), Avelumab (PD‐L1), and Durvalumab (PD‐L1). Based on the working principles, ICI‐induced toxicity typically involves autoimmune disorders,^28^, ^54^, ^555^, ^557^, ^559^ including autoimmune myocarditis.^12^, ^13^, ^14^, ^15^, ^16^, ^17^, ^18^, ^19^, ^20^, ^21^, ^22^, ^23^, ^24^, ^25^, ^26^, ^27^, ^28^, ^93^, ^550^ The importance of ICIs in the heart has been addressed in preclinical data, and the data give some mechanistic insights into the clinical observations of autoimmune myocarditis.^560^, ^561^ In addition, although ICIs are an attractive concept in the therapeutic development of cancer treatment, many cancer patients do not respond to treatments with ICIs, partly because of the lack of tumor‐infiltrating effector T cells^562^, ^563^, ^564^, ^565^ or primary or acquired resistance due to various mechanisms.^565^ The overall response rates against melanoma, bladder cancer, Hodgkin's lymphoma and non‐small cell lung cancer using ICIs is approximately 30%, and complete response rates (eradication of a patient's tumors) are as low as 5%.^566^ Recently, macrophage activation syndrome (MAS), a very severe, new complication of ICIs with a 50% mortality rate, has been reported.^567^ More serious concerns have clearly been raised about the clinical use of ICIs.

Immune cell therapy (T‐cell based therapy) consists of several forms, TILs, cytotoxic T lymphocytes (CTLs), transgenic T‐cell receptor (tgTCR) T cells and CART.^196^, ^568^, ^569^ CART is considered the best‐in‐class example that the genetic engineering of T cells can lead to deep and durable responses in CD19^+^ B cell malignancies.^73^ The receptors allow the modified T cells to attach to specific proteins on the surface of cancer cells, and the modified T cells become activated and attack the cancer cells once bound.^570^ CD19 CART has demonstrated remarkable success in treating hematologic cancers, prominently including acute and chronic B cell leukemias.^571^ Currently, a growing number of clinical trials have focused on solid tumors and targeted surface proteins including carcinoembryonic antigen, diganglioside GD2, mesothelin, interleukin 13 receptor α, human epidermal growth factor receptor 2, fibroblast activation protein, L1 cell adhesion molecule,^568^, ^571^ and melanoma‐associated antigen 3.^32^ However, the clinical results in solid tumors have been much less encouraging.^71^, ^570^ Historically, three generations of CAR constructs have been developed, and the third‐generation CARs are the most recent^196^, ^572^ and contain more than one additional co‐stimulatory molecule. Recently, fourth‐generation CAR T cells redirected for universal cytokine killing (TRUCK) have been described and are designed to express and release transgenic products that accumulate in the targeted tissue.^572^, ^573^, ^574^, ^575^ However, despite tremendous efforts to date CART targeting, CD19 remains the best‐studied example^205^, ^570^, ^576^; CD19 is a cell surface molecule on B cells and B cell malignancies. The FDA granted a ‘breakthrough therapy’ designation to anti‐CD19 CART on 1 July 2014.^196^, ^205^ To date, the challenge for targeted CART continues to be the identification of suitable epitopes to ensure on‐target specificity.^51^, ^53^, ^55^, ^577^ The identification of truly tumor‐specific antigens has become the greatest general challenge in cancer immunotherapy^572^ because true tumor‐specific antigens are rare, and many tumor‐associated antigens are shared by both tumor and normal tissues, resulting in off‐tumor, on‐target toxicity.^53^, ^578^ Cardiotoxicity caused by CART has been reported (Table 3),^30^, ^31^, ^32^, ^33^, ^34^, ^35^, ^36^, ^37^, ^38^, ^39^, ^40^, ^41^, ^42^, ^43^, ^44^ but the causes are diverse, and the mechanisms are not fully understood.^30^, ^31^ In general, the unpredictable specificities of targeted tumor antigens and cytokine release syndrome are recognized as two major factors that are accountable for “on‐target, off‐tumor” toxicity in multiple organs, including cardiotoxicity.^51^, ^53^, ^83^, ^196^, ^579^, ^580^, ^581^, ^582^ Conceptually, neoantigens are the antigens encoded by tumor‐specific mutated genes (known as driver mutations) and specifically expressed in the tumor; thus, neoantigens are theoretically ideal targets for cancer immunotherapy because they are less likely to induce normal tissue toxicity (non‐exclusive antigens).^564^, ^583^ However, the identification of neoantigens depends on targeting “driver” mutations first, and many questions remain unanswered regarding how to precisely define and distinguish between “driver” mutations or clinically actionable mutations (responsive to targeted therapies) from the much larger set of passenger alterations embedded in tumor DNA.^494^ The emerging concept of non‐coding drivers adds additional challenges to precisely define or identify “neoantigens.” In addition, the use of host lymphodepletion‐chemotherapy with immunosuppressive agents (e.g., cyclophosphamide) is required to augment the antitumor effects of CART.^584^ Unfortunately, these concomitant therapies can lead to clinical cardiotoxicity.^585^, ^586^, ^587^, ^588^

The recent experiences with severe, life‐threatening episodes of cardiotoxicity associated with ICIs and CART gives rise to some important concerns that are biologically and clinically relevant for future preclinical studies, oncology trials, and clinical practice to limit the uncontrolled activation of immune responses. Immunotherapies have significant potential, yet there is room for further improvement. The immune system is species‐specific; thus, immunotherapy‐induced toxicity/cardiotoxicity may not be readily predictable in animal models because of the differences in both gene expression and amino acid sequences of peptides derived from homologous proteins.^577^ The insights derived from animal models are limited by significant functional differences of the cardiac and immune systems between animals and humans. For instance, idiosyncratic drug reactions (drug‐ or individual human leukocyte antigen restriction‐specific) are unpredictable, occur only in certain susceptible patients and have a complex dose‐dependent relationship.^589^ Furthermore, the immune system is a complex network of organs, different cell types and molecules that interact among themselves and with other organs, cells, and local and systemic factors. Thus, it is very difficult to predict the behavior of the system from any of its components studied in isolation.^212^ Finally, antigen presentation is not a static event but spatiotemporally dynamic,^564^ which may lead to drug‐mediated immunotoxicity due to unstable immunophenotypes (immunophenotypic drift).^31^, ^55^, ^590^, ^591^ Multi‐organ toxicity resulting from ICIs and CART may reflect the complex scenarios of the immune system in vivo. These problems may be minimized by combining in vitro data with mechanistic mathematical models, which describe intracellular metabolism, fluid‐flow, substrate, hormone, and nutrient distribution and provide the opportunity to mimic the in vivo scenario.^394^, ^577^

## 7 CONCLUSION AND OUTLOOK

Anticancer drug‐induced toxicities occur because agents are not selective, or the targets are not unique to cancer cells. The research gaps resulting from the problematic specificity/selectivity of drugs leave a bottleneck on effective clinical management (curative care) because of uncontrollable toxicities on multiple organs. Improving the specificity/selectivity of drug target selection (druggable molecular targets and tumor‐specific antigens) is considered the single most important factor,^592^ and this strategy will substantially minimize the risk of potential cardiovascular safety liabilities and other organ toxicity. The pharmaceutical industry may have to move toward more selective agents to meet clinical challenges. In line with the mission “oncology drugs still a pipeline priority” (innovation drives the race), as suggested by the US FDA,^593^ numerous approved anticancer drugs and those under investigation have been included in pharmaceutical pipeline databases.^594^, ^595^ Given the explosive rate of new anticancer drug development, there is an urgent need for a synergistic improvement of preclinical studies, clinical trials, pharmacovigilance and post‐marketing surveillance as a whole, including an awareness of biosimilars in oncology. Cardiovascular toxicity has become a very challenging issue during cancer therapy, while consensual guidelines remain lacking for its effective management.^596^ The development of novel therapeutic modalities such as chronotherapy and chronoprevention using natural products of antioxidants should be highly encouraged for both researchers and clinicians to simultaneously address clinical cardiotoxicity and drug efficacy. Furthermore, working with multidisciplinary teams should be considered compulsory to decrease morbidity and mortality from both cardiotoxicity and cancer itself,^597^ and multidisciplinary collaboration is helpful to address the interdisciplinary differences and dilemmas in the meantime. Lastly, kinase inhibitors used in patients with cancer have given a new face to cardiovascular medicine, providing unprecedented insights into the functional roles played by numerous kinases in the cardiovascular system.^2^ This insight will aid in the fundamental understanding of kinase inhibitors and their direct clinical applications in oncology, cardiology, and the cardio‐oncology interface.

## Supporting information

Supplemental References
