# Supplementary material for: Breakthroughs in modern cancer therapy and elusive cardiotoxicity: Critical research‐practice gaps, challenges, and insights
Source: Med Res Rev. 2017 Sep 1;38(1):325–76. doi: 10.1002/med.21463 (PMC5763363; doi:10.1002/med.21463)
Supplement: Supplementary file 1 — Supplemental References [file MED-38-325-s001.doc]

**Supplemental References**

160. Avelumab. <https://www.bavencio.com/en_US/for-patients-and-caregivers.html>. 2017.

161. Avelumab. <https://www.uptodate.com/contents/avelumab-drug-information?source=search_result&search=Bavencio&selectedTitle=1~12>. 2017.

162. Durvalumab. <https://www.uptodate.com/contents/durvalumab-drug-information?source=search_result&search=Imfinzi&selectedTitle=1~11>. 2017.

163. Brigatinib. <https://www.alunbrig.com/>. 2017.

164. Brigatinib. <https://www.uptodate.com/contents/brigatinib-drug-information?source=search_result&search=Brigatinib&selectedTitle=1~8>. 2017.

165. Midostaurin. [https://www.uptodate.com/contents/midostaurin-drug-information?source=search_result&search=Rydapt%20(midostaurin)&selectedTitle=1~11](https://www.uptodate.com/contents/midostaurin-drug-information?source=search_result&search=Rydapt (midostaurin)&selectedTitle=1~11). 2017.

166. Niraparib. [https://www.uptodate.com/contents/niraparib-drug-information?source=search_result&search=Zejula%20(niraparib)&selectedTitle=1~10](https://www.uptodate.com/contents/niraparib-drug-information?source=search_result&search=Zejula (niraparib)&selectedTitle=1~10). 2017.

167. Varricchi G, Marone G, Mercurio V, Galdiero MR, Bonaduce D, Tocchetti CG. Immune Checkpoint Inhibitors and Cardiac Toxicity: An Emerging Issue. Curr Med Chem 2017.

168. Ribociclib. [https://www.uptodate.com/contents/ribociclib-drug-information?source=search_result&search=Kisqali%20(ribociclib)&selectedTitle=1~10](https://www.uptodate.com/contents/ribociclib-drug-information?source=search_result&search=Kisqali (ribociclib)&selectedTitle=1~10). 2017.
